# Dynamic DNA methylation orchestrates cardiomyocyte development, maturation and disease

**DOI:** 10.1038/ncomms6288

**Published:** 2014-10-22

**Authors:** Ralf Gilsbach, Sebastian Preissl, Björn A. Grüning, Tilman Schnick, Lukas Burger, Vladimir Benes, Andreas Würch, Ulrike Bönisch, Stefan Günther, Rolf Backofen, Bernd K. Fleischmann, Dirk Schübeler, Lutz Hein

**Affiliations:** 1 Institute of Experimental and Clinical Pharmacology and Toxicology, University of Freiburg, Albertstrasse 25, 79104 Freiburg, Germany; 2 Hermann Staudinger Graduate School, University of Freiburg, Albertstrasse 21, 79104 Freiburg, Germany; 3 Bioinformatics Group, Department of Computer Science, University of Freiburg, Georges-Köhler-Allee 106, 79110 Freiburg, Germany; 4 Pharmaceutical Bioinformatics, Institute of Pharmaceutical Sciences, University of Freiburg, Hermann-Herder-Strasse 9, 79104 Freiburg, Germany; 5 University Heart Center Freiburg/Bad Krozingen, Department of Congenital Heart Defects and Paediatric Cardiology, University of Freiburg, Hugstetter Strasse 55, 79106 Freiburg, Germany; 6 Friedrich Miescher Institute for Biomedical Research, Maulbeerstrasse 66, 4058 Basel, Switzerland; 7 Swiss Institute of Bioinformatics, Maulbeerstrasse 66, 4058 Basel, Switzerland; 8 European Molecular Biology Laboratory, Genomics Core Facility, Meyerhofstraße 1, 69117 Heidelberg, Germany; 9 Max Planck Institute of Immunobiology and Epigenetics, Stübeweg 51, 79108 Freiburg, Germany; 10 Institute of Physiology I, Life and Brain Center, University of Bonn, Sigmund-Freud-Straße 25, 53127 Bonn, Germany; 11 University of Basel, Petersplatz 1, 4003 Basel, Switzerland; 12 BIOSS Centre for Biological Signalling Studies, University of Freiburg, Schänzlestrasse 18, 79104 Freiburg, Germany

## Abstract

The heart is a highly specialized organ with essential function for the organism throughout life. The significance of DNA methylation in shaping the phenotype of the heart remains only partially known. Here we generate and analyse DNA methylomes from highly purified cardiomyocytes of neonatal, adult healthy and adult failing hearts. We identify large genomic regions that are differentially methylated during cardiomyocyte development and maturation. Demethylation of cardiomyocyte gene bodies correlates strongly with increased gene expression. Silencing of demethylated genes is characterized by the polycomb mark H3K27me3 or by DNA methylation. *De novo* methylation by DNA methyltransferases 3A/B causes repression of fetal cardiac genes, including essential components of the cardiac sarcomere. Failing cardiomyocytes partially resemble neonatal methylation patterns. This study establishes DNA methylation as a highly dynamic process during postnatal growth of cardiomyocytes and their adaptation to pathological stress in a process tightly linked to gene regulation and activity.

## Abstract (web summary)

DNA methylation is essential for proper gene expression, development and genome stability. Here the authors present whole-genome DNA methylation analyses of purified mouse cardiomyocytes from newborn, adult and failing hearts and find highly dynamic patterns between the three phenotypes of cardiomyocytes.

## Graphical abstract

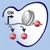

During its development and postnatal life, the heart has to adapt to diverse physiological and pathophysiological needs. Cardiomyocytes arise from progenitors in early development and mature with limited cell division after birth^1^,^2^. The transition from pre- to postnatal life demands a number of contractile and metabolic adaptations to facilitate organ growth in the presence of optimal contractile function^3^,^4^,^5^. Due to its limited regenerative capacity, cardiomyocytes respond to these challenges during development and disease with characteristic gene expression programmes. Several epigenetic processes, including microRNAs^6^, chromatin and histone proteins^7^,^8^,^9^ as well as DNA methylation^10^, have been implicated as modulators of cardiac gene expression in development and disease.

DNA methylation is a stable hallmark of cell type identity and is essential for mammalian development^11^,^12^. It occurs mainly in palindromic CpG dinucleotides. Whereas most regions of the genome are depleted for CpGs, they are clustered in CpG islands. CpG islands mark 70% of annotated mammalian promoters^13^. CpG methylation is essential for proper gene expression, development and genome stability^14^. DNA methylation patterns are maintained during cell division by DNA methyltransferase 1 (DNMT1). *De novo* DNA methylation is mediated by DNMT3A and DNMT3B^15^. Removal of DNA methylation involves oxidation of 5-methyl-cytosine. The key enzymes for this initial step are the recently discovered ten-eleven translocation enzymes TET1-3 (ref. 16). So far there is limited knowledge about the *in vivo* time course of DNA methylation pattern establishment in cardiomyocytes^17^,^18^.

Here we show that DNA methylation is highly dynamic during cardiomyocyte development, postnatal maturation and disease. Demethylated regions in neonatal and adult cardiomyocytes are localized in cell type-specific enhancer regions and in gene bodies of cardiomyocyte genes. Postnatal DNA demethylation correlates with active histone marks and increased gene expression. Repression of demethylated genes is achieved by polycomb-mediated histone H3K27 trimethylation or by *de novo* methylation by DNA methyltransferases DNMT3A/B. Dynamic DNA methylation is important for the perinatal switch in sarcomere protein isoforms and postnatal cardiomyocyte maturation and adaptation.

## Results

### Epigenetic characterization of cardiomyocytes

**Figure 1:**
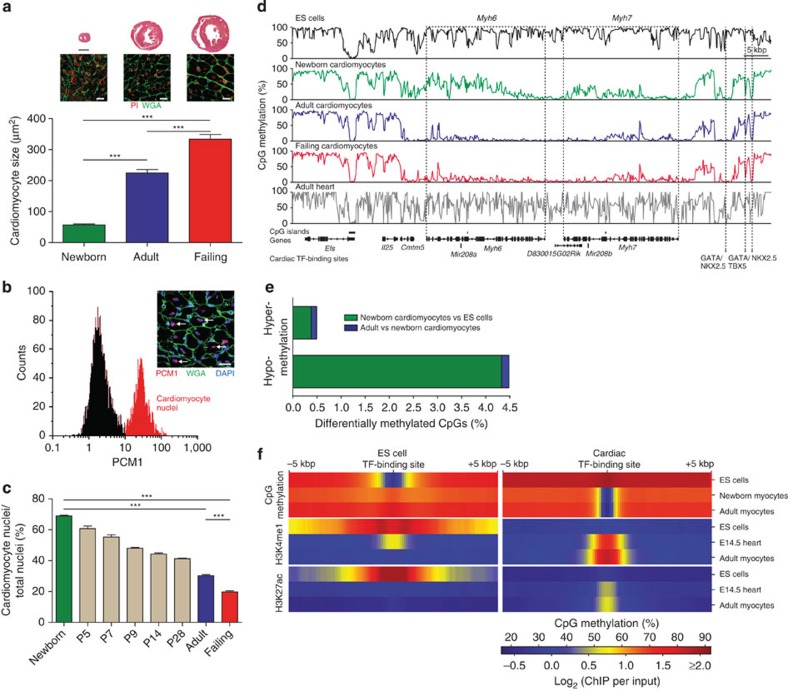
Analysis of DNA methylation in cardiomyocytes isolated from newborn and adult murine hearts. (**a**) Hematoxylin–eosin staining (upper panels; scale bar, 1 mm) of mouse hearts 1 day after birth (newborn), at 8 weeks of age (adult) and 3 weeks after chronic pressure overload in adult mice (failing). WGA-stained ventricular sections (middle panels; scale bar, 20 μm) to determine cardiomyocyte cross-sectional areas (*n*=10 hearts per group). Nuclei were stained with propidium iodide (PI). (**b**) Identification of cardiomyocyte nuclei by PCM1 immunostaining in adult mouse cardiac tissue (insert, arrows; scale bar, 20 μm) and purification of cardiomyocyte nuclei (red) by flow cytometry (histogram). (**c**) Percentage of cardiomyocyte nuclei in ventricular biopsies identified by PCM1 flow cytometry in newborn, adult healthy and failing hearts and at postnatal days 5–28 (*n*=3–6 hearts per group). (**d**) IGV (integrative genomics viewer) traces of CpG methylation of the myosin-6 (*Myh6*) and myosin-7 (*Myh7*) gene region in ES cells, newborn, adult and failing cardiomyocytes and in murine heart tissue. (**e**) Differentially methylated CpGs during fetal and postnatal development in percent of assessed CpGs. (**f**) Density of CpG methylation, histone H3K4me1 and H3K27ac at transcription factor (TF)-binding sites (±5 kbp). Data are shown as mean±s.e.m., ***P<0.001, analysis of variance, Bonferroni *post hoc* test.

To adapt to the requirements of the growing organism, cardiomyocytes increase dramatically in size during physiological postnatal growth (Fig. 1a). In response to cardiac injury, for example, during chronic ventricular pressure overload, cardiomyocytes may initiate further pathological growth (Fig. 1a). To provide insight into the dynamics of DNA methylation in development and disease of cardiomyocytes as a prototypical terminally differentiated cell type, we generated DNA methylomes of newborn, adult healthy and adult failing cardiomyocytes.

Since the heart is a complex tissue with cardiomyocytes contributing only 20–30% of the total cell population^19^, we purified cardiomyocyte nuclei from murine cardiac tissue. An antibody against pericentriolar material 1 (PCM1)^20^ was used to isolate cardiomyocyte nuclei using flow cytometry (fluorescence-activated cell sorting (FACS)) or magnetic-assisted nuclei sorting with very high purity (>97%; Fig. 1b; Supplementary Fig. 1a–c). To validate the specificity of PCM1 staining, we used transgenic mice with cardiomyocyte-specific histone H2B-mCherry expression (Supplementary Fig. 1d). Isolated cardiac nuclei of these animals and wild-type mice were stained with PCM1 antibody or isotype control IgG (immunoglobulin G) and were analysed by FACS (Supplementary Fig. 1e–g). The overlap of mCherry and PCM1-positive nuclei was >95% confirming that PCM1 specifically stains cardiomyocyte nuclei (Supplementary Fig. 1g). Furthermore, a molecular beacon targeting cardiac troponin T type 2 (*Tnnt2*) mRNA stained the major fraction of PCM1-positive nuclei (Supplementary Fig. 1h). Analysis of different developmental and disease stages revealed that the proportion of cardiomyocyte nuclei decreased from 68.7±0.6% in newborn hearts to 29.7±0.5% in adult healthy hearts and to 19.7±0.7% in adult failing hearts (mean±s.e.m., *n*=6; Fig. 1c; Supplementary Fig. 1i–k). DNA from these purified cardiomyocyte nuclei was bisulfite converted and DNA methylomes were obtained at base-pair resolution using deep sequencing. Three independent experiments with high reproducibility (Supplementary Fig. 2) resulted in high-coverage methylomes (Supplementary Fig. 3; Supplementary Table 1). DNA methylomes from murine embryonic stem (ES) cells^21^ and from total cardiac tissue^10^ were used for comparison.

To gain further insight into the epigenetic landscape, we generated maps for four histone modifications (H3K4me1, H3K4me3, H3K27ac and H3K27me3) from purified cardiomyocyte nuclei (Supplementary Table 2). To generate matching cardiomyocyte-specific transcriptomes, hearts were digested enzymatically, vital cardiomyocytes were sorted by FACS and cardiomyocyte mRNA was subjected to RNA sequencing.

### Differential CpG methylation

Visual inspection of two prototypical cardiomyocyte genes, α- and β-myosin heavy chains (*Myh6* and *Myh7*), revealed a progressive CpG demethylation in cardiomyocytes purified from newborn as compared with adult and failing hearts (Fig. 1d). Almost complete demethylation of gene body CpGs of *Myh6* and *Myh7* was apparent in purified cardiomyocyte nuclei but could not be detected in cardiac tissue^10^, which contains several additional cell types (Fig. 1d). To further validate this finding, methylation of CpGs of the cardiomyocyte-specific gene encoding for the sarcoplasmic reticulum Ca^2+^ ATPase (SERCA2A, gene *Atp2a2*) was analysed by pyrosequencing (Supplementary Fig. 4). *Atp2a2* methylation levels greatly differed between myocyte and non-myocyte cell fractions with intermediate levels in cardiac tissue (Supplementary Fig. 4a–d).

In total, we identified 79,655 differentially methylated regions (DMRs) with an average size of 840 bp when comparing adult healthy cardiomyocytes and undifferentiated ES cells (Supplementary Fig. 5). This amounted to 5% of all CpGs in the mouse genome (Fig. 1e). Ninety percent of these CpGs were hypomethylated and 10% were hypermethylated in cardiomyocytes versus ES cells (Fig. 1e). Remarkably, a substantial number of CpGs were differentially methylated between newborn and adult cardiomyocytes (6,436 DMRs; Fig. 1e). These data indicate that differential CpG methylation was not restricted to cell type specification during embryonic development, but extended well into the postnatal period of cardiomyocytes. Genomic annotation of cardiomyocyte DMRs showed a predominance for intragenic and CpG island-flanking regions (Supplementary Fig. 6).

### Hypomethylated regions with cardiomyocyte enhancer signature

Previous studies have identified transcription factor-binding sites and histone marks, which are characteristic for enhancers in demethylated regions^10^,^21^,^22^. This prompted us to analyse the enrichment of transcription factor (TF)-binding motifs in DMRs. Demethylated regions in ES cells (Fig. 1f) were significantly enriched (*P*<10^−20^, hypergeometric test) for binding sites of stem cell transcription factors including OCT4 (gene symbol *Pou5f1*), NANOG and others (Supplementary Data 1). In contrast, demethylated regions of adult cardiomyocytes were significantly enriched (*P*<10^−50^, hypergeometric test) for TF motifs of known cardiac transcription factors including MEF2C, GATA1-4 and others (Fig. 1f; Supplementary Fig. 7a–c; Supplementary Data 1). These TF included the recently identified master regulators of cellular identity of ES cells or hearts, respectively^23^. In the demethylated state, TF-binding regions were found to be enriched for histone modifications (H3K4me1, H3K27ac), which are typical for active *cis*-regulatory sites^21^,^24^ (Fig. 1f). In line with being regulatory, these DMRs were located within distal regulatory regions of genes characteristic for ES cells or cardiomyocytes, respectively (Supplementary Fig. 7d,e).

### Genic CpG demethylation affects specific gene programmes

**Figure 2:**
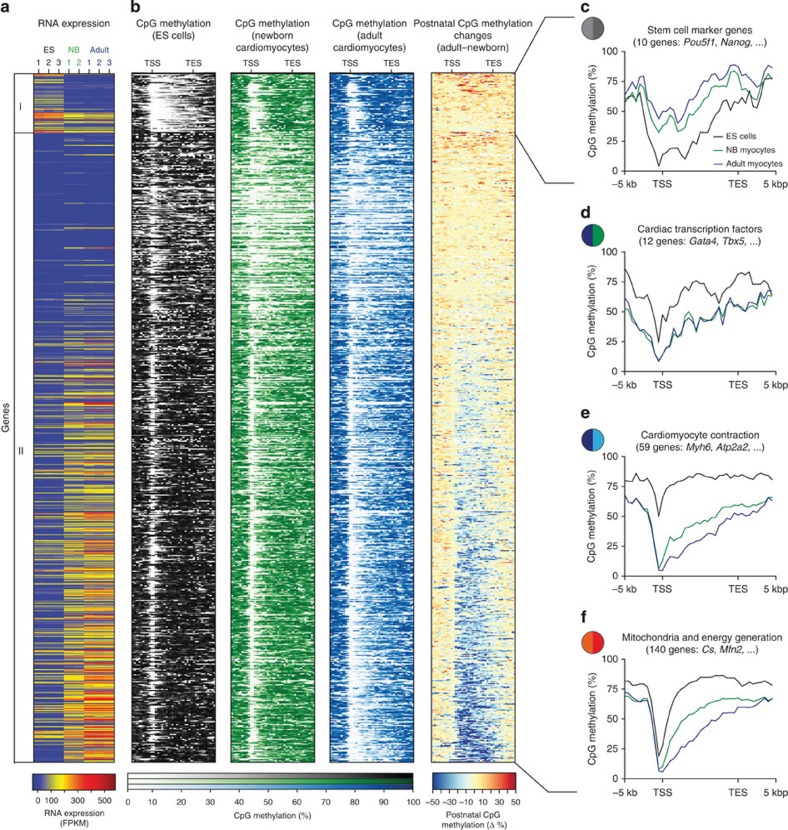
Differentially methylated gene bodies in neonatal and adult cardiomyocytes versus ES cells. (**a**,**b**) Heat maps of gene expression (**a**) and CpG methylation (**b**) of gene bodies and flanking regions (±5 kbp) with differential methylation in neonatal and adult cardiomyocytes versus ES cells. Group I, genes with hypermethylated gene bodies in adult cardiomyocytes versus ES cells; group II, genes with hypomethylated gene bodies. (**c**–**f**) CpG methylation profiles of grouped genes with selected biological function and flanking regions (±5 kbp). Coloured circles refer to Supplementary Figs 8 and 9. TSS, transcription start site; TES, transcription end site.

Since many sites of dynamic DNA methylation occurred within genic regions (Supplementary Fig. 6), we explored this aspect in more detail (Fig. 2a,b). At 765 genes, genic DMRs extended to more than 25% of the entire gene body (Fig. 2a,b). In 78 cases, gene bodies were demethylated in ES cells, yet they were methylated to a higher degree in cardiomyocytes (Fig. 2b, group I; Supplementary Data 2). Gene ontology analysis of this group revealed a predominance of genes involved in embryonic pattern specification and morphogenesis (Supplementary Fig. 8) containing known pluripotency factors like *Pou5f1* and *Nanog* (Fig. 2c; Supplementary Data 2). However, the inverse case with 687 genes, which were demethylated in cardiomyocytes versus ES cells, was much more prevalent (Fig. 2a,b, group II). This group was highly enriched in genes involved in cardiac transcriptional regulation, cardiac development, muscle contraction and energy supply (Fig. 2d–f; Supplementary Fig. 9). Genes that were further demethylated during postnatal maturation of cardiomyocytes were involved in cardiac contraction or mitochondrial function (Fig. 2e,f).

### Genic CpG methylation correlates with gene expression

**Figure 3:**
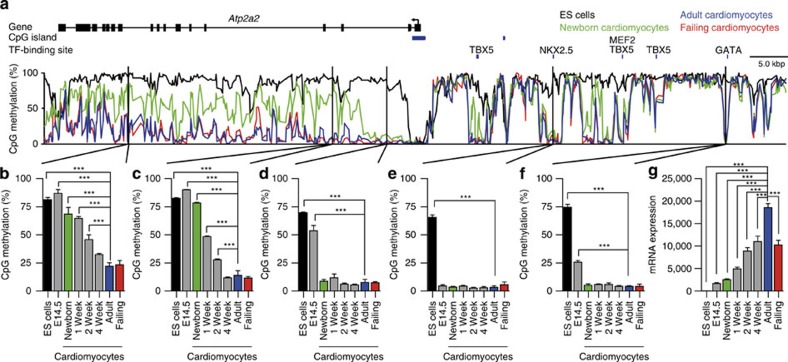
CpG methylation and gene expression of the *Atp2a2* gene. (**a**–**f**) Gene body demethylation of the *Atp2a2* gene and transcription factor-associated DMRs were assessed during fetal and postnatal development in purified cardiomyocyte nuclei by pyrosequencing (assays I (**b**), II (**c**), III (**d**), V (**e**), VI (**f**); Supplementary Fig. 4a). (**g**) Gene expression of *Atp2a2* during fetal and postnatal development (**a**–**g**). Data are shown as mean±s.e.m., *n*=3–9 per group, ***P<0.001 compared with adult, analysis of variance, Bonferroni *post hoc* test.

To gain more insight into the kinetics of DNA methylation, we analysed the time course of gene body demethylation of the *Atp2a2* gene by pyrosequencing (Fig. 3a–f; Supplementary Fig. 4). Demethylation of *Atp2a2* in cardiomyocytes was initiated at the 5′ end and progressed towards the 3′ region of the gene (Fig. 3a–d). While the 5′ end of the gene body and upstream TF motifs were already demethylated at birth, the 3′ region lost its CpG methylation by adulthood (Fig. 3b–f). Gene body demethylation correlated with increased expression of *Atp2a2* during postnatal cardiomyocyte maturation (Fig. 3g). Adult failing cardiomyocytes expressed lower levels of *Atp2a2* than adult control myocytes (Fig. 3g) without significant changes in gene body methylation (Fig. 3b–g).

**Figure 4:**
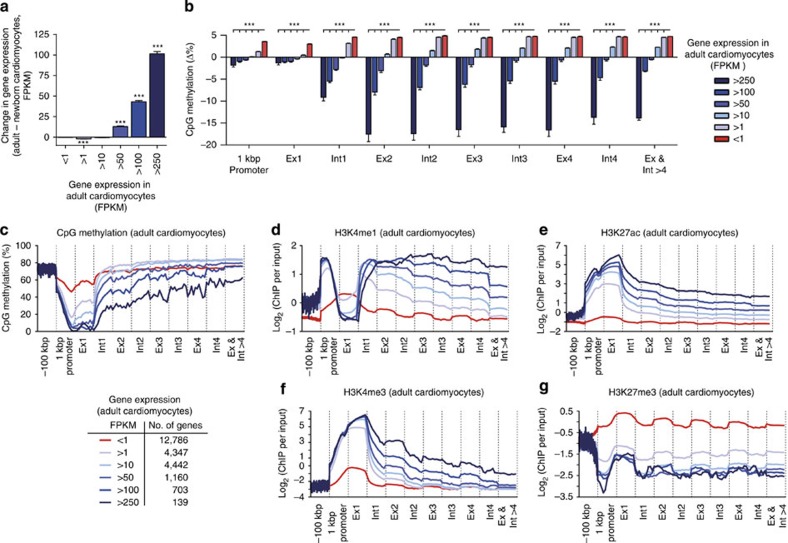
Correlation of gene expression, CpG methylation and histone modifications in genic regions. (**a**) Gene expression in adult versus newborn cardiomyocytes was classified according to adult expression levels. Data are shown as mean±s.e.m., *n*=3, ***P<0.001 adult versus newborn expression, analysis of variance (ANOVA), Bonferroni *post hoc* test. (**b**) Changes in DNA methylation of promoters, exon and intron regions between adult and neonatal cardiomyocytes according to adult gene expression levels. Data are shown as mean±s.e.m., *n*=3, ***P<0.001, ANOVA, Bonferroni *post hoc* test. (**c**–**g**) Averaged CpG methylation levels and histone enrichments in genic, promoter (1 kbp) and upstream (100 kbp) regions of adult cardiomyocytes. Genes were grouped according to gene expression levels in adult cardiomyocytes. Ex, exon; Int, intron.

To search for a possible relationship between gene body CpG methylation status and gene expression, we ranked all genes according to their expression level in adult cardiomyocytes and determined the changes in gene expression and DNA methylation after birth (Fig. 4a,b). Those genes that showed the greatest increase in expression after birth (Fig. 4a; expression increase >250 fragments per kilobase of transcript per million reads, FPKM) were most strongly demethylated at their gene bodies during postnatal development (Fig. 4b). Genes with demethylation of gene bodies after birth were involved in several metabolic processes (Supplementary Fig. 10). Inactive genes showed a higher level of CpG methylation at the promoter and the first exon when compared with expressed genes (Fig. 4c). CpG methylation of the first exon and the remainder of the gene was inversely correlated with gene expression (Fig. 4c). Actively expressed genes were decorated with H3K4me1, H3K27ac and H3K4me3 and they lacked H3K27me3 (Fig. 4d–g). In contrast, gene bodies of inactive genes showed abundant H3K27me3 in addition to high levels of CpG methylation (Fig. 4c,g). These genes were grouped into distinct functional categories (Supplementary Fig. 11). Taken together, these data demonstrate that a demethylation wave runs through the gene body of actively transcribed genes. This wave is initiated at the 5′ end of cardiomyocyte genes, becomes further extended until adulthood and correlates with gene expression.

### Polycomb-mediated silencing of early developmental genes

**Figure 5:**
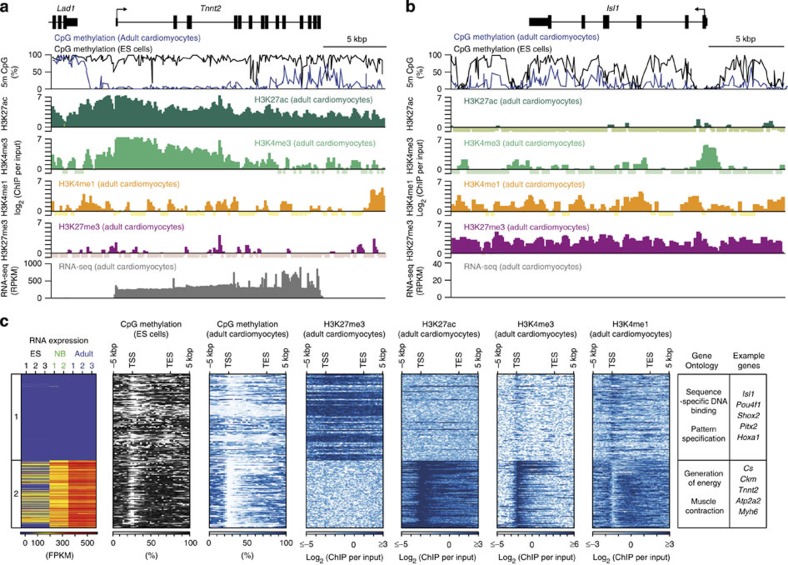
Repression of genes by the polycomb mark H3K27me3. (**a**,**b**) IGV (integrative genomics viewer) traces of CpG methylation of the cardiac troponin T type 2 (*Tnnt2*) gene (**a**) and the *Isl1* gene (**b**) in adult cardiomyocytes and ES cells and traces of histone modifications and RNA expression. (**c**) Heat maps of gene expression, CpG methylation and histone modifications of genes demethylated in adult cardiomyocytes as compared with ES cells. Displayed genes (derived from Fig. 2a, group II) had either very low (<1 FPKM, group 1) or high gene expression (>250 FPKM, group 2) in adult cardiomyocytes (Supplementary Data 2). Right panel, enriched gene ontology terms (*P*<10^−15^, hypergeometric test, Bonferroni step down correction) and representative genes. TSS, transcription start site; TES, transcription end site; 5 m CpG, GpG methylation.

The group of genes, which were demethylated in their gene bodies in cardiomyocytes as compared with ES cells (Fig. 2a,b, group II), contained genes that were known to be expressed during cardiac development but showed no significant expression in adult cardiomyocytes. Thus, we asked whether specific histone marks characterize demethylated genes that are inactive in postnatal cardiomyocytes. First, two prototypic genes with either high levels of expression in adult cardiomyocytes (*Tnnt2*) or expression restricted to early cardiac development (*Isl1*) were analysed (Fig. 5a,b). Visual inspection of the *Tnnt2* locus revealed a demethylated gene body in adult cardiomyocytes, which was covered with active chromatin marks H3K27ac, H3K4me3 and H3K4me1 (Fig. 5a). The cardiac differentiation factor *Isl1* gene, which is expressed at embryonic day (E) 8.5^25^ but not in postnatal cardiomyocytes, remained demethylated until adulthood too. Thus, DNA methylation was not restored on gene inactivation. However, the *Isl1* gene was covered with the repressive chromatin mark H3K27me3 in newborn (Supplementary Fig. 12) and adult cardiomyocytes (Fig. 5b). The gain of this repressive mark set by the enzymatically active subunit enhancer of zeste homologue 2 (EZH2) of the polycomb repressive complex 2 (PRC2) was not restricted to this gene but rather typical for demethylated genes that were not expressed (<1 FPKM; Fig. 2a, group II) in adult cardiomyocytes (Fig. 5c, group 1). Affected genes were expressed either in early embryogenesis, such as the transcription factors of the Hox cluster^26^, or at early stages of cardiac development, such as *Isl1 and Pitx2* (ref. 27).

We asked whether the affected genes were already marked by H3K27me3 in developmental stages. Analysis of H3K27me3 in newborn cardiomyocytes as well as reanalysis of previously published data sets^28^ were performed. These data revealed enrichment of H3K27me3 in embryonic hearts at E12.5 (ref. 28) and in newborn cardiomyocytes (Supplementary Fig. 12a). In addition, EZH2 was found to be localized at these genes in embryonic hearts at E12.5 (ref. 28) (Supplementary Fig. 12a). Recently, the functional importance of PRC2 for proper heart development was shown by cardiac ablation of EZH2 (refs 28, 29). Remarkably, a subset of genes including *Cdkn2a*, *Isl1* and the previously described polycomb target genes *Six1* and *Pax6* (refs 28, 29), which were decorated by H3K27me3, showed increased gene expression on EZH2 ablation^28^,^29^ (Supplementary Fig. 12b–e; Supplementary Table 3). Interestingly, these alterations were more evident in E12.5 hearts as compared with right ventricles of adult hearts^28^,^29^ (Supplementary Table 3). In contrast, none of the CpG-demethylated and actively transcribed genes (>250 FPKM; Fig. 2a, group II) were marked by H3K27me3, but all of these genes were decorated with H3K27ac, H3K4me3 and H3K4me1 (Fig. 5c, group 2).

### Postnatal *de novo* DNA methylation requires DNMT3A/B

**Figure 6:**
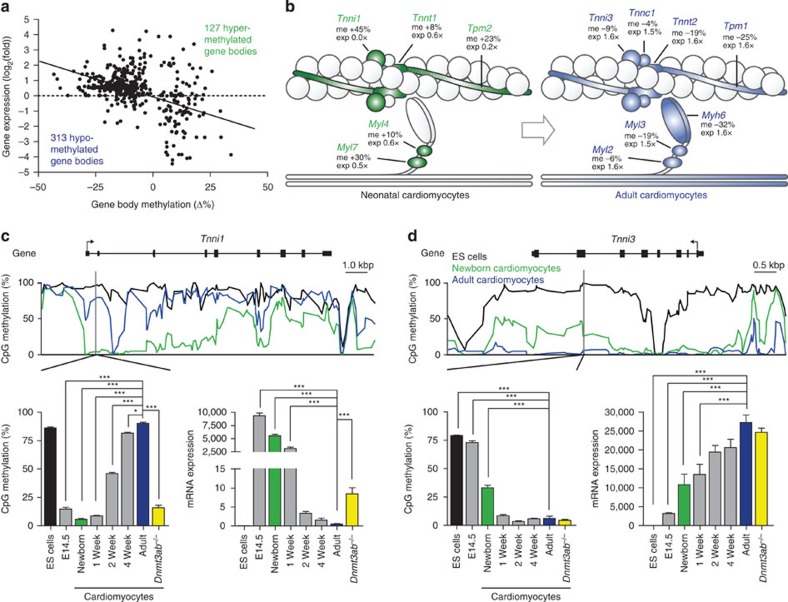
Repression of genes by *de novo* DNA methylation. (**a**) Inverse correlation of DNA methylation changes in cardiac myocytes and gene expression changes during postnatal development (*P*<0.0001, linear regression). (**b**) Schematic view of a cardiac myofilament at birth (left) and in adult heart (right). Sarcomere components, which are differentially methylated in their genes during postnatal development from birth to adulthood, are filled with green or blue colour. Green colour indicates genes that have low levels of CpG methylation at birth and are significantly higher methylated in adult cardiomyocytes. Blue colour indicates genes with lower methylation in adult versus neonatal cardiomyocytes. Values next to sarcomere components indicate the difference in gene expression (exp) and CpG methylation (me) in adult versus newborn cardiomyocytes. (**c**,**d**) DNA methylation traces of troponin I genes *Tnni1* (**c**) and *Tnni3* (**d**) in ES cells, newborn and adult cardiomyocytes (upper panels). Time course of genic DNA methylation (left graphs) and mRNA expression (right graphs). Data are shown as mean±s.e.m., *n*=3–4 per group, *P<0.05, ***P<0.001 compared with adult, analysis of variance, Bonferroni *post hoc* test; *Dnmt3ab*^−/−^ versus adult control, Student’s *t*-test.

Postnatal maturation of cardiomyocytes was not only accompanied by demethylation of gene bodies but also by *de novo* DNA methylation (Supplementary Data 2). Overall, 127 genes gained methylation and 313 lost methylation of their gene bodies during the postnatal period (Fig. 6a). These changes in postnatal DNA methylation of gene bodies correlated inversely with gene expression (Fig. 6a; Supplementary Data 2). Genes that were hypermethylated in cardiomyocytes after birth were involved in myocyte contraction (Fig. 6b), cardiac morphogenesis, cell differentiation and other processes (Supplementary Fig. 11). Functional adaptation of the heart during the postnatal period includes isoform switches in the expression of sarcomere proteins^4^,^5^. As an example, we analysed DNA methylation and expression of the fetal and adult troponin I isoforms that are encoded by *Tnni1* and *Tnni3* genes, respectively (Fig. 6b–d). The gene body of *Tnni1* was demethylated at birth and it was *de novo* methylated in adult cardiomyocytes (Fig. 6b,c). Postnatal *de novo* DNA methylation correlated with repression of *Tnni1* expression (Fig. 6c). In contrast, the adult troponin I isoform *Tnni3* was demethylated and turned on in its expression after birth (Fig. 6b,d). To test whether postnatal DNA methylation and gene repression were mediated by *de novo* DNA methyltransferases 3A/B, mice with cardiac myocyte-specific deletion of the *Dnmt3a* and *Dnmt3b* genes were generated and analysed (Fig. 6c,d). Ablation of DNMT3A/B expression completely prevented postnatal *de novo* methylation of *Tnni1* and partially relieved repression of this gene (Fig. 6c). Similarly, postnatal DNA methylation of the sarcomere components *Tpm2* and the glucose transporter GLUT1 gene (*Slc2a1*) was mediated by DNMT3A/B (Supplementary Fig. 13). Thus, isoform switching of sarcomere proteins during postnatal life was accompanied by *de novo* methylation of fetal genes and demethylation of adult isoforms (Fig. 6b).

As postnatal cardiomyocyte maturation is accompanied by cell cycle arrest^30^,^31^, we analysed postnatal CpG methylation dynamics of all genes, which were contained in the gene ontology group ‘cell cycle’ (GO:0007049; Supplementary Fig. 14). Twenty-one out of these 1,279 genes in this group were differentially methylated during postnatal development (Supplementary Fig. 14a). Most of the differentially methylated cell cycle-associated genes showed opposite changes of DNA methylation and RNA expression after birth (Supplementary Fig. 14b) as further illustrated for *Ccnd3* and *Bmp7* (Supplementary Fig. 14c,d). Both genes showed their highest expression levels at E14.5. Postnatal *de novo* DNA methylation of *Ccnd3* and *Bmp7* was absent in cardiomyocytes from *Dnmt3a/b*-deficient mice. However, in these cases, *Dnmt3a/b* ablation did not affect gene expression (Supplementary Fig. 14c,d).

Analysis of expression of the group of cell cycle-associated genes revealed that 502 genes were differentially expressed in adult versus newborn cardiomyocytes (Supplementary Fig. 15a). Thus, we asked whether polycomb-mediated gene silencing is involved in the regulation of these cell cycle genes. However, postnatally repressed cell cycles genes were not decorated with the repressive mark H3K7me3 and did not show increased genic CpG methylation (Supplementary Fig. 15b).

### DNA methylation changes after chronic pressure overload

**Figure 7:**
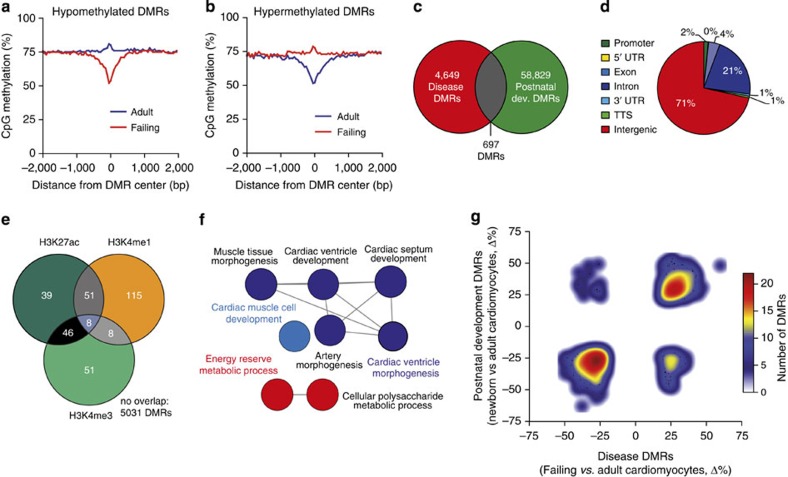
Developmental pattern of CpG methylation in cardiomyocytes in experimental murine heart failure. (**a**,**b**) Overlay plot of CpG methylation in DMRs and surrounding regions of cardiomyocytes isolated from healthy or failing adult hearts displaying hypomethylated (**a**) and hypermethylated (**b**) DMRs. (**c**) Venn diagram showing partial overlap (‘common DMRs’) of disease DMRs (differential methylation between failing and healthy cardiomyocytes) and postnatal DMRs (differential methylation between healthy newborn and adult cardiomyocytes). (**d**) Genome annotation of DNA regions with differential CpG methylation between failing and healthy adult cardiomyocytes. (**e**) Intersection of disease DMRs with H3K27ac, H3K4me1 and H3K4me3 peaks in adult cardiomyocytes. (**f**) Functional annotation of common DMRs in postnatal development and disease (hypergeometric test, Bonferroni step down correction, *P* value per GO term <0.02). (**g**) Density plot of common DMRs showing direction of methylation changes in postnatal development and failure. TTS, transcription termination site (region −100 bp to+1 kbp); UTR, untranslated region.

To explore how the cardiac methylome changes in heart disease, we analysed differential CpG methylation of cardiomyocyte nuclei that were purified from failing murine hearts after chronic left ventricular pressure overload (Supplementary Fig. 16a–e). Compared with developmental alterations, the observed changes in CpG methylation were smaller in scale (Fig. 7a,b). We therefore adjusted the cutoff criteria for DMR detection to 20% differential methylation. Using these less-stringent criteria, we identified 5,346 disease-associated DMRs (Fig. 7c). These DMRs were mostly intergenic (Fig. 7d). Disease-associated DMRs (5.9%) showed an overlap with enhancer or promoter regions marked by H3K27ac, H3K4me1 and H3K4me3 in adult cardiomyocytes (Fig. 7e). Disease-associated DMRs overlapping with postnatal DMRs were adjacent to genes involved in cardiac muscle cell development, cardiac morphogenesis and energy metabolism (Fig. 7f), indicating adaptation of DNA methylation in disease-relevant regions. Methylation levels of these disease-associated DMRs partially resembled the newborn CpG methylation pattern (Fig. 7g).

## Discussion

**Figure 8:**
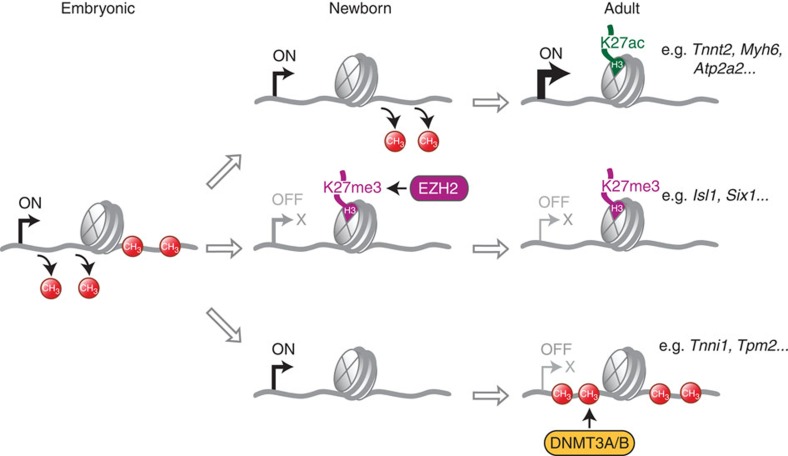
Dynamics of DNA methylation and histone modification in genes bodies of cardiomyocyte genes during development. Gene bodies of cardiomyocyte genes are sequentially demethylated from embryonic stages (left part, E14.5, data from Fig. 3) until newborn (middle part) and adult stages (right part). The degree of gene body demethylation correlates with histone H3K27ac marks and high expression levels of cardiomyocyte genes (upper trace, data from Figs 1, 3, 5 and 6; Supplementary Fig. 4). Demethylated genes, which are transiently expressed during embryonic development, can be repressed by EZH2-mediated H3K27 trimethylation (middle trace, data from Fig. 5; Supplementary Fig. 12). Demethylated genes may also be repressed by *de novo* methylation, which is mediated by DNMT3A/B (lower trace, data from Fig. 6; Supplementary Fig. 14).

The present study resolves the DNA methylome of cardiomyocytes at base-pair resolution from embryonic development to postnatal maturation and disease. For this purpose, cardiomyocyte nuclei were highly purified from frozen heart tissue by FACS and magnetic-assisted sorting. One of the most intriguing features of the cardiomyocyte DNA methylome is a demethylation wave running through gene bodies of cardiomyocyte genes, which orchestrates with *de novo* DNA methylation and activation of polycomb marks to shape the epigenome of maturing cardiomyocytes after birth (Fig. 8).

Bisulfite sequencing has uncovered distinct patterns of DNA methylation in different genomic contexts, including promoters, gene bodies and regulatory elements^12^. We identified several distinct features of DNA methylation also in cardiomyocytes. When compared with ES cells as a model for an undifferentiated, pluripotent cell type, cardiomyocytes contained short regions of low DNA methylation, which showed characteristics of *cis*-regulatory elements. Tissue-specific DMRs were previously identified for a large number of human and murine cell and tissue types^10^,^22^. Demethylated regions contained binding motifs for tissue-specific transcription factors and contained histone marks that are characteristic for poised (H3K4me1) or active (H3K27ac) enhancers^10^,^22^. Our data demonstrate that demethylation of enhancers was already established in newborn cardiomyocytes and contained cardiac-specific transcription factor motifs that are characteristic for super-enhancers of the cardiac lineage^23^. These cardiomyocyte-specific sites were characterized by H3K4me1 marks and part of them also contained H3K27ac, thus identifying them as poised or active enhancers^24^, respectively. Previous studies have suggested that transcription factor activity could be a key factor for active DNA demethylation of enhancer regions^21^,^32^. Thus, the DNA methylomes presented in this study provide comprehensive enhancer maps of cardiomyocytes during development and maturation, which extends the previous studies in cardiac tissues.

As a second characteristic feature of the cardiomyocyte DNA methylome, we identified large demethylated regions that covered entire gene bodies of large cardiomyocyte genes, for example, the myosin heavy-chain locus or the *Atp2a2* gene (Fig. 8, upper trace). Some of the longest demethylated regions were identified in the genes encoding for the cardiac ryanodine receptor (*Ryr2*, 180 kbp), the α_1C_-subunit of the L-type Ca^2+^ channel (*Cacna1c*, 162 kbp) or titin (*Ttn*, 121 kbp; Supplementary Data 2). The significance of DNA methylation in gene bodies is still only partially understood^12^ and studies have shown both positive or negative correlations between genic DNA methylation and gene expression. In neurons^33^, hematopoietic stem cells^34^ and other lineages^35^, demethylation of regions containing the transcription start and gene bodies was identified in highly expressed genes. In contrast, a positive correlation between CpG methylation and gene expression was identified in human ES cells^36^, mouse oocytes^37^ or B lymphocytes^38^. In the present study, gene bodies were demethylated starting at the 5′ end and extending towards the 3′ region during postnatal maturation of cardiomyocytes. Genic demethylation correlated well with active histone marks and increased expression of cardiomyocyte genes.

However, not all genes that were demethylated in cardiomyocytes versus ES cells were also expressed in postnatal cardiomyocytes. More than one hundred genes were DNA demethylated and decorated by trimethylation of H3K27 (Fig. 8, middle trace). This histone mark is associated with polycomb-mediated gene repression, and EZH2 is one of the core proteins of the PRC2 in the heart^28^,^29^. Deficiency of EZH2 in cardiomyocytes caused lethal heart malformations^28^ or postnatal cardiomyopathy^29^, depending on the time of EZH2 ablation. Binding of EZH2 and H3K27me3 could be identifed at E12.5 and H3K27me3 marks persisted at the same loci in newborn and adult cardiomyocytes. Demethylated genes that were transiently expressed during embryonic development, for example, *Isl1* (ref. 25) and *Pitx2* (ref. 27), were stably repressed by H3K27me3. However, deletion of *Ezh2* led to reactivation of *Isl1*, *Pitx2* and other developmental genes^28^,^29^. It is interesting to note that H3K27me3 was specifically associated with repressed and demethylated genes in cardiomyocytes. This finding is supported by previous studies that have detected H3K27me3-mediated gene repression in genomic regions lacking DNA methylation^34^,^35^. Most recently, it was shown that polycomb may selectively be recruited to unmethylated DNA sequences and transcriptional activity or CpG methylation prevented polycomb binding and function^39^.

As a second mechanism for repression of demethylated cardiomyocyte genes, we identified *de novo* CpG methylation mediated by DNA methyltransferases 3A/B (Fig. 8, lower trace). Genes that were hypo- or hypermethylated in cardiomyocytes after birth were involved in multiple physiological processes including the transition from fetal to adult sarcomeric gene expression. To adapt to the specific demands of pre- versus postnatal life, several protein isoforms of the sarcomere are switched during the perinatal phase by an unknown mechanism^5^. Indeed, we found the slow skeletal troponin I isoform (*Tnni1*) to be repressed by postnatal DNA methylation, which was mediated by DNMT3A/B. Cardiomyocyte-specific ablation of DNMT3A/B completely prevented *Tnni1* methylation after birth and partially reactivated gene expression. The perinatal switch from fetal to adult sarcomere protein isoforms has been associated with important aspects of myocyte development and contractile function^4^,^5^. In particular, the decrease in Ca^2+^ sensitivity of the sarcomere has been linked with the transition from slow skeletal (*Tnni1*) to cardiac troponin I (*Tnni3*)^4^. In addition to the fetal sarcomere isoforms, genes involved in the fetal to adult transition of energy metabolism^3^ and several other process were differentially methylated in their gene body after birth. These findings suggest that dynamic gene methylation may play an important role in cardiomyocyte switching and maturation after birth.

To determine possible alterations in the DNA methylome during cardiac disease, we have subjected mice to 3 weeks of cardiac pressure overload. Constriction of the aortic arch resulted in ventricular hypertrophy, interstitial fibrosis and pulmonary oedema, thus partially resembling the phenotype of chronic human heart failure. While DNA methylation in failing cardiomyocytes returned to a fetal methylation pattern, the observed changes were less promiment and abundant compared with physiological postnatal myocyte maturation. Thus, future studies will be important to determine whether the human cardiomyocyte DNA methylome shows similar features during development and in disease. Recent studies have investigated DNA methylation in human cardiac tissue biopsies from patients with chronic heart failure of different etiologies^17^,^18^. Both studies described distinct signatures of DNA methylation in failing versus non-failing hearts. The present study describes a path to assign such alterations to a specific cell type in the heart. Future studies will be important to uncover the DNA methylomes of other cardiac cell types, for example, fibroblasts, endothelial cells, immune cells and so on, to determine their epigenetic contribution to cardiac development and disease.

The present study establishes DNA methylation in cardiomyocytes as dynamic and reversible during development, postnatal maturation and disease. Future studies unravelling the signals and mechanisms involved in shaping the cardiac DNA methylome will be essential for a better understanding of cardiac myocyte biology.

## Methods

### Animal procedures

All animal procedures were approved by the responsible animal care committee (Regierungspräsidium Freiburg, Germany) and they conformed to the Guide for the Care and Use of Laboratory Animals published by the National Academy of Sciences, 2011. Mice with cardiac myocyte-specific ablation of DNMT3A/B expression were generated by crossing *Dnmt3a*^*flox*^ and *Dnmt3b*^*flox*^ mice^40^ with mice expressing cre recombinase under control of the cardiac *Mlc2a* promoter^41^. Male mice of the indicated ages were dissected to obtain cardiac tissues. To induce cardiac pressure overload by transverse aortic constriction for 3 weeks, male mice (8 weeks old) were anaesthetized with 2% (vol) isoflurane in oxygen. After thoracotomy, a 7.0 silk suture was placed around a 27-G hypodermic needle to constrict the transverse aorta^42^.

### ES cells

Murine ES cells, which were originally derived from blastocysts of mixed 129—C57BL6 background^21^, were maintained in ES cell medium (DMEM, 15% fetal calf serum (FCS), non-essential amino acids, 10 ng ml^−1^ leukaemia inhibiting factor, 0.1% β-mercaptoethanol) on gelatine-coated plates^21^.

### Echocardiography

Echocardiography was performed using a Vivid 7 Dimension (GE Healthcare, Munich, Germany) echocardiograph equipped with a 14-MHz transducer^42^. Parasternal short-axis views were used for M-mode analysis. Enddiastolic and endsystolic left ventricular inner diameters (LVIDd, LVIDs) were measured and fractional shortening (FS) and ejection fraction (EF) were calculated using the following equations: FS=(LVIDd—LVIDs)/LVID, EF=(LVIDd^3^−LVIDs^3^)/LVIDd^3^ (ref. 42).

### Histology

Hearts were fixed with 4% paraformaldehyde in phosphate-buffered saline (PBS), embedded in paraffin, cut into 3 μm slices and stained with haematoxylin–eosin, Sirius-red or fluorescent wheat germ agglutinin (WGA; Alexa Fluor 488 conjugate, Invitrogen, Karlsruhe, Germany). Nuclei were counterstained with propidium iodide (Sigma, Munich, Germany). For immunohistochemical staining, hearts were snap frozen, embedded in optimum cutting temperature (OCT) medium (Tissue-Tek), cut into 3 μm slices and stained with an antibody against PCM1 (1:1,000, HPA023370, Sigma) in combination with a Cy3-labelled anti-rabbit antibody (1:1,000, Invitrogen). Glycocalyx was stained with WGA (Alexa Fluor 488 conjugate, Invitrogen) and nuclei were counterstained with DAPI (4′,6-diamidino-2-phenylindole, Invitrogen).

### Flow cytometric analysis and sorting of cardiomyocyte nuclei

Cardiomyocyte nuclei were isolated from cardiac tissue^20^ and were stained by an antibody directed against PCM1 (1:1,000, HPA023370, Sigma) and an Alexa488-labelled secondary anti-rabbit antibody (1:1,000, Invitrogen) or a molecular beacon targeting mRNA^43^ of *Tnnt2* (Supplementary Data 3; 50 nM) for 30 min. Nuclei were identified by 7-aminoactinomycin D (7-AAD) (1:500, Invitrogen). For analysis of αMHC-H2B-mCherry transgenic mice, an Alexa647-labelled secondary anti-rabbit antibody (1:1,000, Invitrogen) and for nuclei-staining DAPI (1:1,000, Invitrogen) was used. Nuclei were analysed or sorted by flow cytometry (CyFlow Space, Partec, Münster, Germany; BD Influx cell sorter, BD Biosciences, Heidelberg, Germany; Bio-Rad S3 cell sorter, Bio-Rad Laboratories, Munich, Germany).

### Magnetic-assisted sorting

Magnetic-assisted cell sorting (MACS) of myocyte nuclei was performed after isolation and staining of nuclei with a PCM1 antibody (1:1,000, HPA023370, Sigma). Nuclei were centrifuged and resuspended in 80 μl MACS buffer. Anti-rabbit IgG MicroBeads (20 μl; Miltenyi, Bergisch Gladbach, Germany) were added and incubated for 15 min at 4 °C. MACS buffer (900 μl) was added and the nuclei suspension was applied to an M column (Miltenyi), washed with 3 ml MACS buffer and eluted with 1 ml MACS buffer after removal of the magnet. The eluate was applied to another M column, washed again and eluted in 1 ml elution buffer (1 mM EDTA in PBS). Sorting efficiency was determined by staining aliquots before and after cell separation with an Alexa488-labelled secondary anti-rabbit antibody (1:1,000, Invitrogen) and by flow cytometric analysis (CyFlow Space, Partec).

### Isolation of cardiomyocytes for transcriptome analysis

Adult and neonatal hearts were dissociated to obtain a single-cell suspension^44^,^45^. Adult hearts were dissociated by retrograde perfusion with Tyrode’s solution supplemented with 25 mM butanedione monoxime, 2 mM CaCl_2_, 0.8 mg ml^−1^ collagenase B (Roche, Mannheim, Germany), 0.4 mg ml^−1^ hyaluronidase (Sigma) and 3 μg ml^−1^ trypsin (Sigma) for 12 min. Hearts were gently dissected and enzymatic digestion was stopped by addition of Tyrode’s buffer supplemented with 5% FCS, 25 mM butanedione monoxime and 2 mM CaCl_2_. Newborn hearts were dissociated by several rounds of digestion with trypsin (Gibco) in Hank’s Balanced Salt Solution (HBSS, Life Technologies). Digestion was stopped by resuspension in HBSS containing 4% FCS. Cell suspensions of adult and neonatal hearts were kept at 4 °C until sorting. Sorting of cardiomyocytes from cardiac cell suspensions was carried out at 4 °C using a Bio-Rad S3 cell sorter. Cardiomyocytes and non-cardiomyocytes were visualized using the cell-permeable nucleid acid stain Vybrant DyeCycle Ruby (Life Technologies). Non-viable cells were identified by counterstaining with the cell-impermeable nucleid acid stains Sytox Green or 7-AAD (Life Technologies). Cardiomyocytes were discriminated from other cardiac cells by high forward scatter (FSC) signals. FSC pulse width was used to exclude doublets from sorting. Gates were selected to sort viable cardiomyocytes directly into RLT lysis buffer (Qiagen, Hilden, Germany). To confirm the gating strategy, small aliquots were permeabilized with 0.1% saponin (Sigma) and stained with antibodies against cardiac troponin I (ab47003, Abcam) and α-actinin (At7811, Sigma) in combination with secondary Alexa Fluor-coupled antibodies (Life Technologies).

### External data sets

Previously published MethylC-seq data from murine ES cells (GSE30206)^21^ and murine adult heart (GSM1051154)^10^ were downloaded, mapped and analysed in the same way as the methylomes generated in this study. Previously published chromatin immunoprecipitation-sequencing (ChIP-seq) data for H3K27ac (GSM851290) and H3K4me1 (GSM851287) for embryonic heart (E14.5)^46^ and ES cells (GSM594577 and GSM594579)^24^ were reanalysed. ChIP-seq data for H3K27me3 and EZH2 for E12.5 hearts (GSE29994) were reanalyzed^28^. RNA-seq data of *Ezh2* mutant mice and corresponding controls were reanalyzed (GSE29992)^28^,^29^.

### Whole-genome bisulfite sequencing

MethylC-seq was performed as described^36^. In brief, 1 μg of genomic DNA was extracted from purified nuclei (AllPrep DNA/RNA Mini Kit, Qiagen). DNA-seq libraries were prepared with methylation adapters (ILMN ME-100-0010, Illumina, San Diego, USA), bisulfite conversion was carried out with the EZ DNA methylation kit (D5001, Zymo Research, Freiburg, Germany) for 16 h and final PCR was performed with the KAPA Uracil+ system (16 cycles, KAPAbiosystems KK2801). All libraries were sequenced on a paired-end 101-bp sequencing run (Illumina HiSeq 2000).

### CpG methylation analysis

All bioinformatic tools used in this study were integrated into the Galaxy web server^47^,^48^,^49^. The generated data format is compliant with the BED file format. Sequencing reads were trimmed (Phred quality score±20) and mapped using Bismark^50^ (bowtie 1 (ref. 51), mouse genome assembly mm9) and PCR duplicates were removed with SAMtools^52^. For further analysis, we developed a set of bioinformatic tools (Methtools; https://github.com/bgruening/methtools), including differential methylation analysis. Results for the sense and antisense strand were combined after calling of CpG methylation values. Only CpGs with a minimal coverage of 4 were included in further analysis steps. In case of ES cell data^21^, SNPs between C57BL/6J and 129S5 mouse strains were filtered. Methylation values were smoothed using a running average of three consecutive CpGs. For determination of DMRs of two methylomes (DMRs), data sets were intersected to exclude CpGs determined in only one data set using Bedtools^53^. A sliding window approach was used to identify DMRs. Regions displaying a mean CpG methylation delta ≥40% over five CpGs between methylomes were selected as DMRs. Additional exclusion criteria were a minimal difference of 10% for each individual CpG and a maximal distance of 1,000 bp between adjacent CpGs. Selected regions were extended stepwise until they failed to fulfil the cutoff criteria and were then reported as DMRs. In case of disease-associated DMRs, a mean delta of 20% or greater was accepted. Differential gene body methylation between ES cells and adult cardiomyocytes was identified by DMRs covering >25% of a gene body with a minimal DMR length of 1 kbp. For comparison of newborn and adult cardiomyocytes, the cutoff criterion was >5% of the gene body. Genes smaller than 500 bp were excluded from all analyses. The Integrative Genomics Viewer^54^, Bioconductor^55^ and deepTools^56^ were used for visualization.

### Motif analysis and binding site prediction

Location of transcription factor-binding sites and motif enrichments within DMRs were determined by the Homer tool^57^ using default parameters. The known motifs used in this study were derived from the Homer tool and were validated by ChIP-seq. Motifs lacking this experimental proof were omitted. HAND1/HAND2 motifs were derived from JASPAR.

### Pyrosequencing

Genomic DNA (500 ng) was extracted from MACS- or FACS-purified nuclei (AllPrep DNA/RNA Mini Kit, Qiagen) and was bisulfite converted with the EZ DNA Methylation Kit (D5001, Zymo Research). The concentration of converted DNA was adjusted to 5 ng μl^−1^ and DNA was amplified with the PyroMark PCR Kit (Qiagen) using a three-primer approach with a universal biotinylated primer^58^. PCR products were checked by gel electrophoresis, pyrosequenced using PyroMark Gold Q24 Reagents (Qiagen) on a PyroMark Q24 instrument^59^ and quantified with the PyroMark Q24 software (Qiagen). For each assay, a standard curve of 0, 25, 50, 75 and 100% methylated DNA was measured. The GenomePlex Complete WGA Kit (Sigma) was used to generate unmethylated DNA and the CpG methyltransferase M.Sssl (NEB, Frankfurt, Germany) was used to generate methylated DNA. Primer sequences are listed in Supplementary Data 3.

### Annotation of *cis*-regulatory regions

Prediction of functional significance of regions was performed using the GREAT tool^60^. The basal gene regulatory domain was defined as the region starting 5 kbp upstream and ending 1 kbp downstream of the TSS. The gene regulatory domain was extended in both directions up to 50 kbp to the nearest gene’s basal domain.

### Gene ontology analysis

ClueGO^61^ was used to find over-represented GO terms in the categories ‘biological process’, ‘cellular component’ and ‘molecular function’. Benjamini–Hochberg correction was performed for multiple testing-controlled *P* values. Significantly enriched terms were functionally grouped and visualized. The GO term connectivity threshold was adjusted to >0.3 (kappa value). The highest significant term of the group was displayed as leading term.

### CpG island and genomic annotation

Random sequences from MethylC-seq-assessable genomic regions were selected to analyse genome representation of genomic features and CpG islands. Genomic feature annotation was performed using Homer tools^57^. CpG island annotation^62^ and UCSC gene prediction annotation^63^ were downloaded from UCSC.

### Gene expression analysis

Polyadenylated RNA was isolated from 500 ng of total RNA with magnetic beads (NEBNext Poly(A) mRNA Magnetic Isolation Module, NEB). Libraries were constructed (NEBNext Ultra RNA Library Prep Kit for Illumina, NEB) and size selection was performed with AMPure XP Beads (Beckman Coulter, Krefeld, Germany). Thirteen PCR cycles were used for library amplification and were sequenced on a HiSeq 2500 (50 bp, Illumina, San Diego, USA). RNA-seq data were mapped to the mm9 genome assembly using Tophat^64^. FPKM values were calculated by Cufflinks^65^ as an estimate of transcript expression. Differential gene expression was calculated using Cuffdiff^65^. The analysis was performed with default parameters and the UCSC gene annotation^63^ as a reference. Quantitative real-time PCR was carried out as described^66^. Primer sequences are listed in Supplementary Data 3.

### ChIP-sequencing

ChIP-seq was performed as described with modifications^67^. Lysis buffers for preparation of nuclei were supplemented with 10 mM sodium butyrate (Sigma). MACS-purified nuclei were fixed with 1% freshly prepared paraformaldehyde (Sigma) for 2 min at room temperature. Fixation was stopped by adding glycine to a final concentration of 125 mM. Nuclei were washed three times with PBS and were lysed in SDS buffer (50 mM Tris–HCl, pH 8.0, 10 mM EDTA, 1% SDS) supplemented with protease inhibitors (Roche Complete Protease Inhibitor Cocktail, Roche). Chromatin was sheared using a Bioruptor (Diagenode, Liege, Belgium) with 30 high-energy cycles (30 s on/30 s off) to an average size of 200–400 bp. Lysates were diluted 1:10 with buffer (22 mM Tris–HCl, pH 8.0, 165 mM NaCl, 2.2 mM EDTA, 1.1% Triton X-100) and were precleared with 0.1% bovine serum albumin for 2 h at 4 °C. Antibodies were preincubated with Protein A Dynabeads (Invitrogen) for 2 h at 4 °C. For immunoprecipitation, either 5 μg of chromatin was incubated with antibodies directed against H3K4me3 (2 μg, pAb-003-050, Diagenode) and H3K27ac (2 μg, ab4729, Abcam, Cambridge, UK) overnight at 4 °C or 2.5 μg of chromatin was incubated with an anti-H3K4me1 antibody (1 μg, ab8895, Abcam) or an anti-H3K27me3 antibody (1 μg, pAb-069-050, Diagenode). Immunocomplexes were washed for 10 min each with buffer 1 (20 mM Tris–HCl, pH 8.0, 150 mM NaCl, 2 mM EDTA, 1% Triton X-100, 0.1% SDS), buffer 2 (20 mM Tris–HCl, pH 8.0, 500 mM NaCl, 2 mM EDTA, 1% Triton X-100, 0.1% SDS), buffer 3 (20 mM Tris–HCl, pH 8.0, 250 mM LiCl, 2 mM EDTA, 1% IGEPAL CA-630, 1% sodium deoxycholate) and three times with TE buffer (20 mM Tris–HCl, pH 8.0, 2 mM EDTA) at 4 °C. Elution from beads was carried out by shaking in elution buffer (100 mM NaHCO_3_, 1% SDS) at 1,400 r.p.m. at room temperature for 1 h. DNA was treated with 50 ng μl^−1^ RNAse A (Sigma) for 30 min at 37 °C and crosslinks were reversed by adding 0.5 μg μl^−1^ proteinase K (AppliChem, Darmstadt, Germany) and incubation at 65 °C for 5 h. DNA was purified using phenol/chloroform followed by ethanol precipitation at −20 °C overnight. DNA was quantified using Quant-iT PicoGreen dsDNA Reagent (Invitrogen). Sequencing libraries were prepared from 5 ng DNA with the NEBNext ChIP-seq Library Prep Master Mix Set for Illumina (NEB) with 15 PCR cycles and sequenced on a HiSeq 2500 (50 bp, Illumina, San Diego, USA).

### ChIP-seq data analysis

ChIP-seq reads were mapped to the mm9 genome assembly by Bowtie2 (ref. 68). For read coverage of input and histone modifications, the genome was binned. The number of reads per bin was counted and normalized to the total number of mappable reads (reads per kilobase per million mapped reads, RPKM). The enrichment of histone modifications in a given genomic region was calculated as the log2 ratio of ChIP RPKM and input RPKM. Peak calling was performed using MACS2 (ref. 69).

## Author contributions

R.G. and S.P. designed and performed the experiments. T.S. performed surgery and physiology. V.B. and U.B. performed sequencing and A.W. performed FACS. B.A.G. developed bioinformatic tools and B.A.G., R.G. and S.P. performed computational analysis. L.B., S.G., R.B. and B.K.F. interpreted the data and edited the manuscript. L.H. conceived the project and L.H., D.S., R.G. and S.P. wrote the manuscript.

## Additional information

**Accession codes:** DNA methylomes, ChIP-Seq and RNA-seq files have been submitted to the NCBI BioSample databases under accessions SRP033288 (MethylC-seq), SRP033385 (ChIP-seq) and SRP033386 (RNA-seq).

**How to cite this article:** Gilsbach, R. *et al.* Dynamic DNA methylation orchestrates cardiomyocyte development, maturation and disease. *Nat. Commun.* 5:5288 doi: 10.1038/ncomms6288 (2014).

## Supplementary Material

Supplementary Information Supplementary Figures 1-16, Supplementary Tables 1-3 and Supplementary References

Supplementary Data 1 Analysis of transcription factor (TF) enrichment of hypo- and hypermethylated DMRs in adult cardiomyocytes as compared with ES cells.

Supplementary Data 2 Life of genes with differential gene body methylation

Supplementary Data 3 Primer sequences.
