# Supplementary material for: Dynamic DNA methylation orchestrates cardiomyocyte development, maturation and disease
Source: Nat Commun. 2014 Oct 22;5:5288. doi: 10.1038/ncomms6288 (PMC4220495; doi:10.1038/ncomms6288)
Supplement: Supplementary Information — Supplementary Figures 1-16, Supplementary Tables 1-3 and Supplementary References [file ncomms6288-s1.pdf]

Supplementary Figure 1: Isolation of cardiomyocyte nuclei from murine hearts

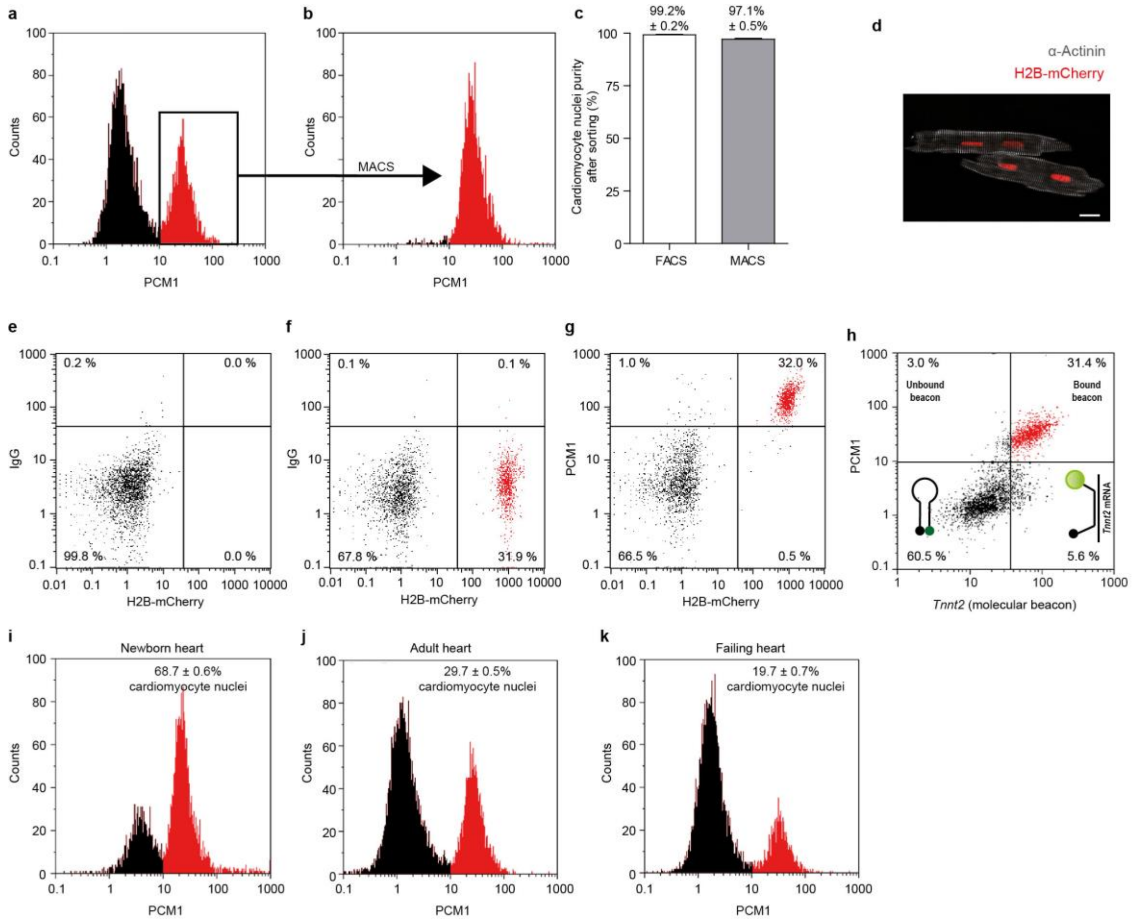

Suppl. Fig. 1: Flow cytometric analysis (FACS) of cardiomyocyte nuclei with an antibody against pericentriolar material 1 (PCM1, a-c, g-k), a cardiac myocyte-specific histone H2B-mCherry transgene (d-g) or a molecular beacon targeting mRNA of cardiac troponin T type 2 (*Tnnt2*, h). **a, b,** FACS histograms of cardiomyocyte nuclei identified by PCM1 (red) and non-myocyte nuclei (black) from adult murine hearts before (a) and after purification of nuclei with magnetic-assisted sorting (MACS) with PCM1 antibody (b). **c,** Purity of PCM1-positive cardiomyocyte nuclei after FACS- or MACS-based isolation of cardiomyocyte nuclei (n = 4-10). **d-g,** Analysis of adult heart nuclei from mice expressing H2B-mCherry under control of the cardiomyocyte-specific αMHC-promoter (H2B-mCherry). **d,** mCherry fluorescence in nuclei of cardiomyocytes from H2B-mCherry mice (overlay of white, α-actinin immunostaining; red, H2B-mCherry fluorescence; scale bar 20 µm). **e-g,** FACS analysis of cardiac nuclei isolated from H2B-mCherry mice. Nuclei of wild-type mice were stained with isotype control IgG as a negative control (e). Cardiac nuclei from H2B-mCherry mice showed nuclei with high mCherry fluorescence (red) (f). PCM1-positive and mCherry positive nuclei overlapped (red) (g). **h,** Overlap of PCM1 staining with a molecular beacon targeting *Tnnt2* mRNA which is specifically expressed in cardiomyocytes. The *Tnnt2* beacon bound to its target mRNA was identified by its 6-carboxyfluorescein fluorescence (right symbol). Fluorescence of unbound beacons was quenched by BHQ (black hole quencher, left symbol). **i-k,** Representative FACS histograms and quantification of PCM1-positive

cardiomyocyte nuclei from newborn (i), adult healthy (j) and failing (k) murine hearts (*n* = 6 per group). Data are displayed as mean values ± s.e.m., *n* = 6.

Supplementary Figure 2: Correlation of CpG methylation between experimental replicates

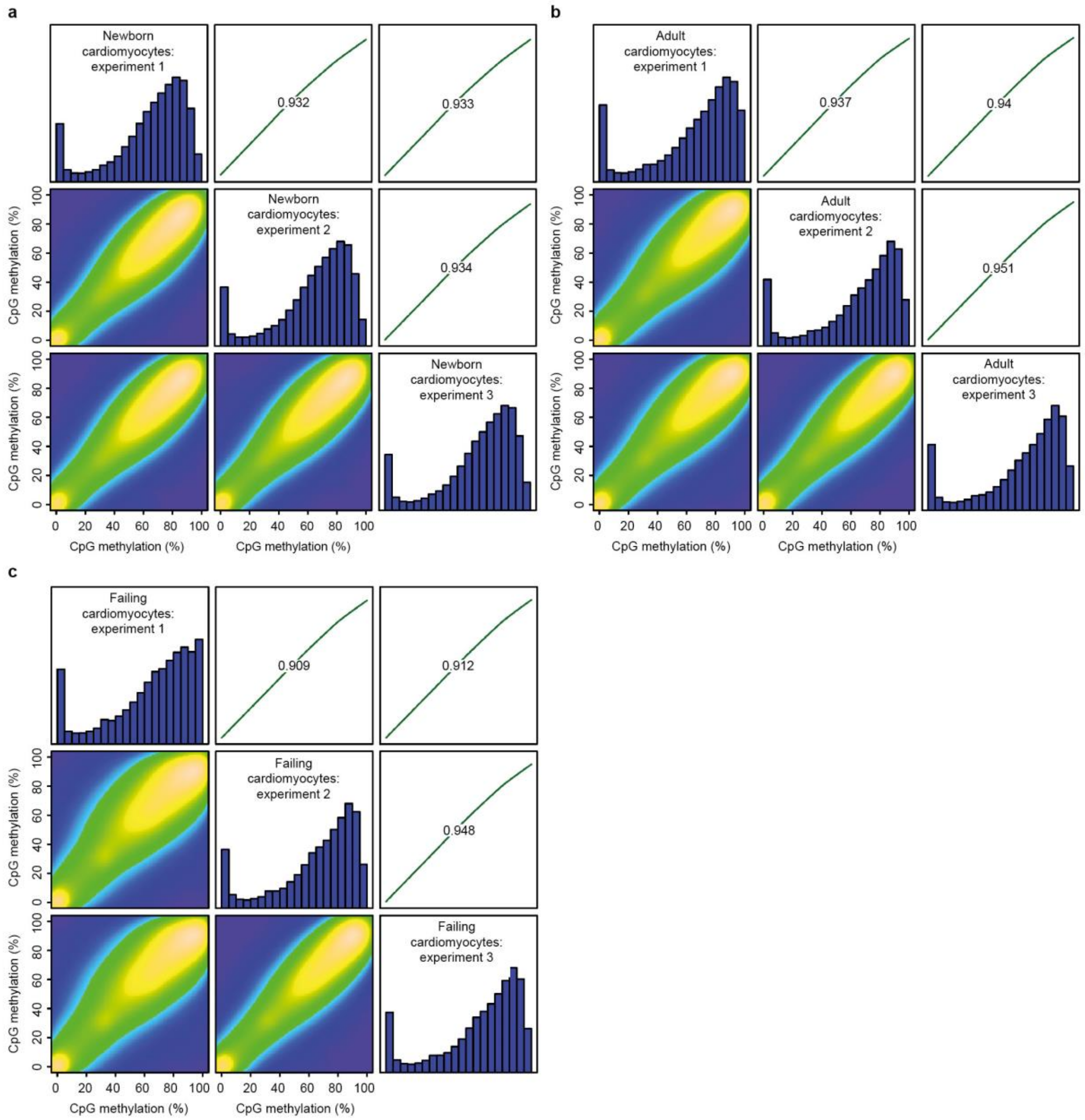

Suppl. Fig. 2: Histograms of CpG methylation, scatter plots and Pearson correlation coefficients for three experimental replicates of newborn myocytes (a), adult healthy (b) and failing cardiomyocytes (c). Data represent CpGs with a minimal coverage of 10.

Supplementary Figure 3: MethylC-seq sequencing statistics

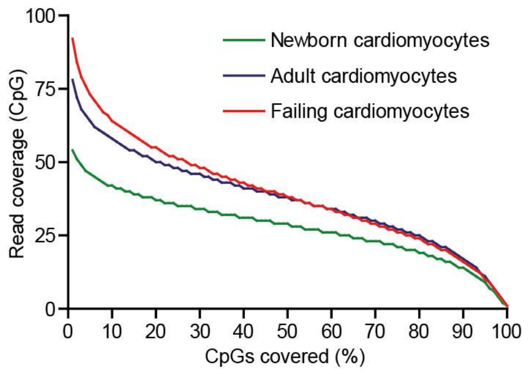

Suppl. Fig. 3: Read coverage of CpG methylation analysis for neonatal, adult healthy and adult failing cardiomyocytes.

Supplementary Figure 4: CpG methylation of the *Atp2a2* locus in cardiomyocytes and non-myocytes

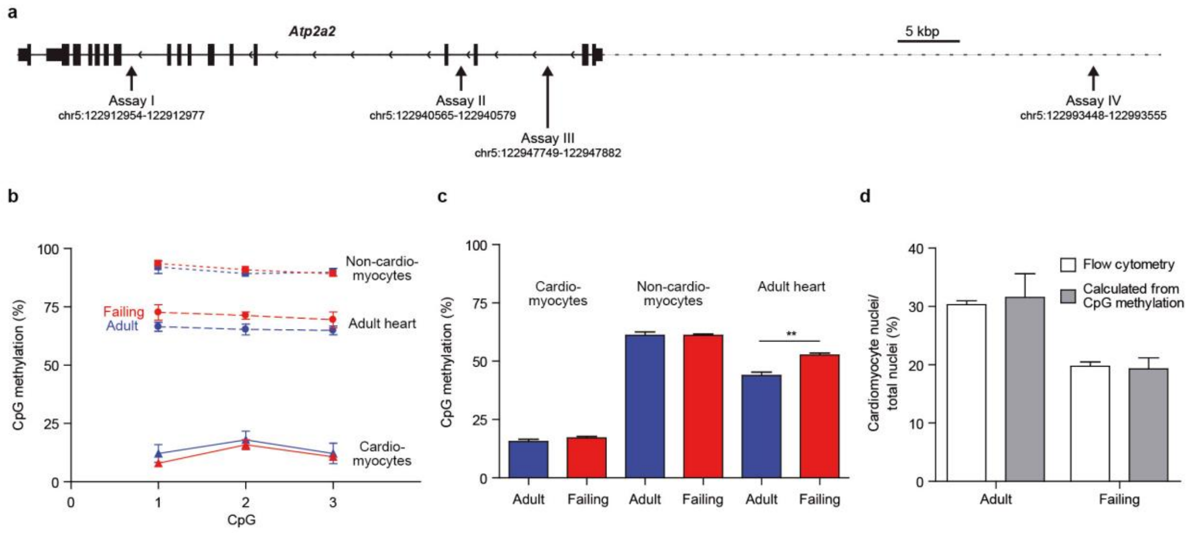

Suppl. Fig. 4: a, Schematic representation of the murine *Atp2a2* gene and position of the primers used for pyrosequencing to assess CpG methylation (assays I-IV, for primers see Supplementary Tab. 6). **b,** Methylation of three CpGs (assay II) in *Atp2a2* in healthy and failing adult cardiomyocytes (PCM1-positive nuclei) and non-myocytes (PCM1-negative nuclei) as compared with heart tissue (*n* = 3-4 samples per group). **c,** Average methylation of 4 CpGs (assay IV) upstream of *Atp2a2* in healthy and failing adult cardiomyocytes (PCM1-positive nuclei) and non-myocytes (PCM1-negative nuclei) as compared with heart tissue (*n* = 3-4 samples per group, ***P* < 0.01, ANOVA, Bonferroni post hoc test). **d,** Fraction of cardiomyocyte nuclei in adult and failing hearts determined either by flow cytometry using PCM1 staining or by calculation of CpG methylation levels in cardiomyocytes, non-myocytes and cardiac tissue (average methylation of 11 CpGs using assays I-IV, *n* = 3-6). Data are shown as mean values ± s.e.m.

Supplementary Figure 5: Size distribution of differentially methylated regions (DMRs)

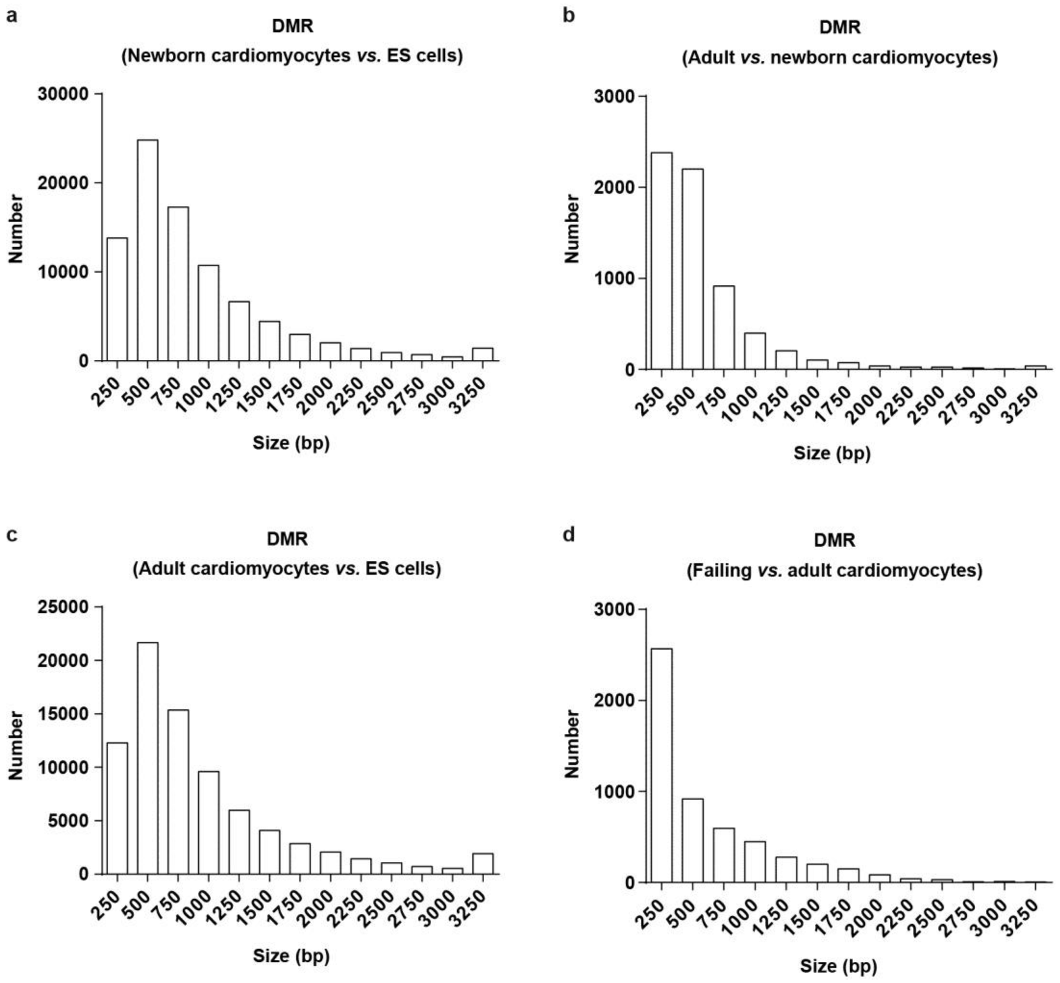

Suppl. Fig. 5: a-d, Histograms of DMR size distributions between neonatal, adult and failing cardiomyocytes and ES cells (bin size 250 bp).

Supplementary Figure 6: DMR annotation

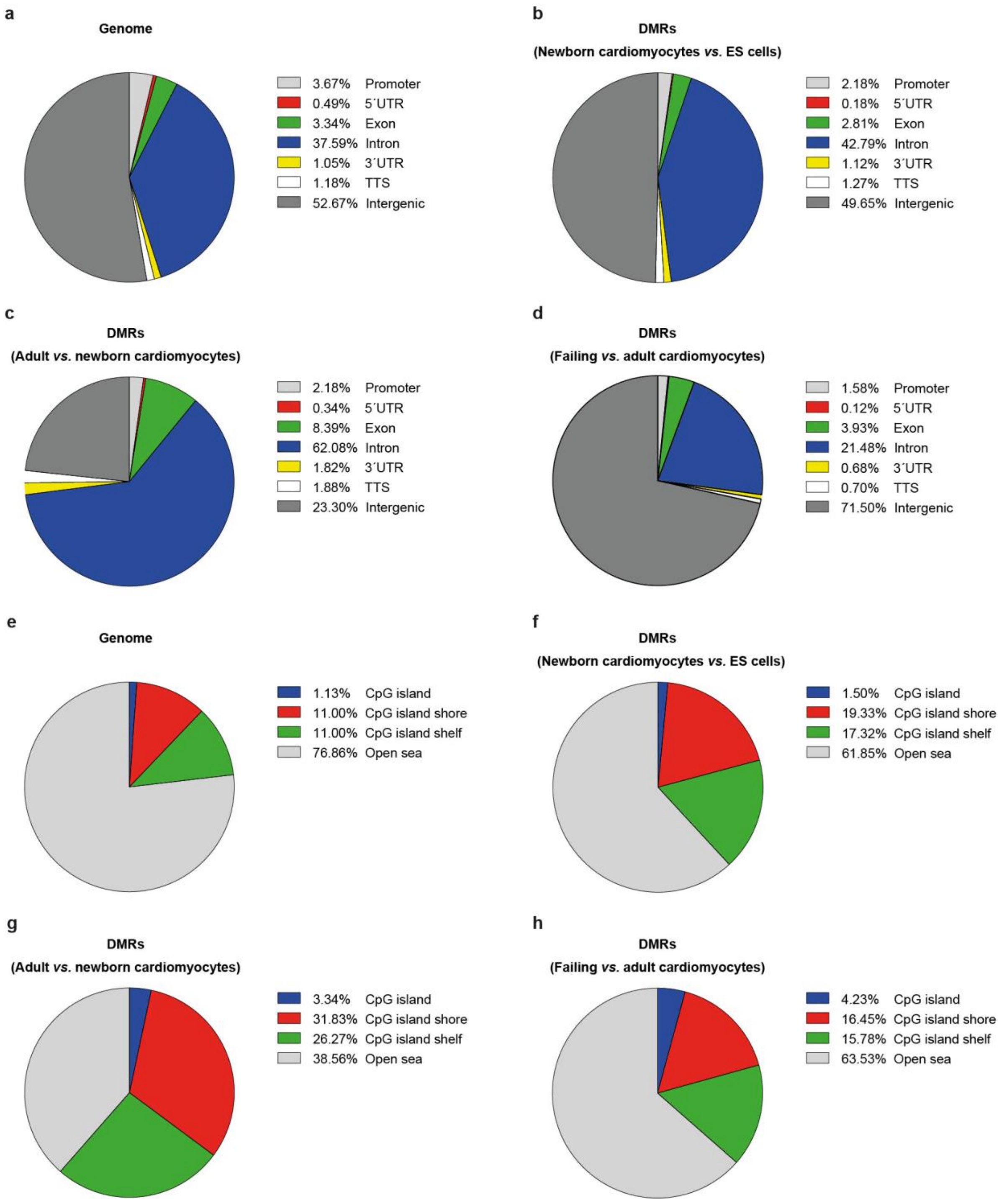

Suppl. Fig. 6: a-d, Genomic annotation of the average genome (a) and DNA regions with differential CpG methylation (DMRs) between newborn, adult and failing cardiomyocytes *vs.* ES cells (b-d). **e-h,** CpG island annotation of the average genome (e) and DNA regions with differential CpG methylation (DMRs) between newborn, adult and failing cardiomyocytes *vs.* ES cells (f-h). Abbreviations: UTR, untranslated region; TTS, transcription termination site (region -100 bp to + 1 kbp).

Supplementary Figure 7: DNA methylation at cardiac transcription factor binding sites in differentially methylated regions (DMRs)

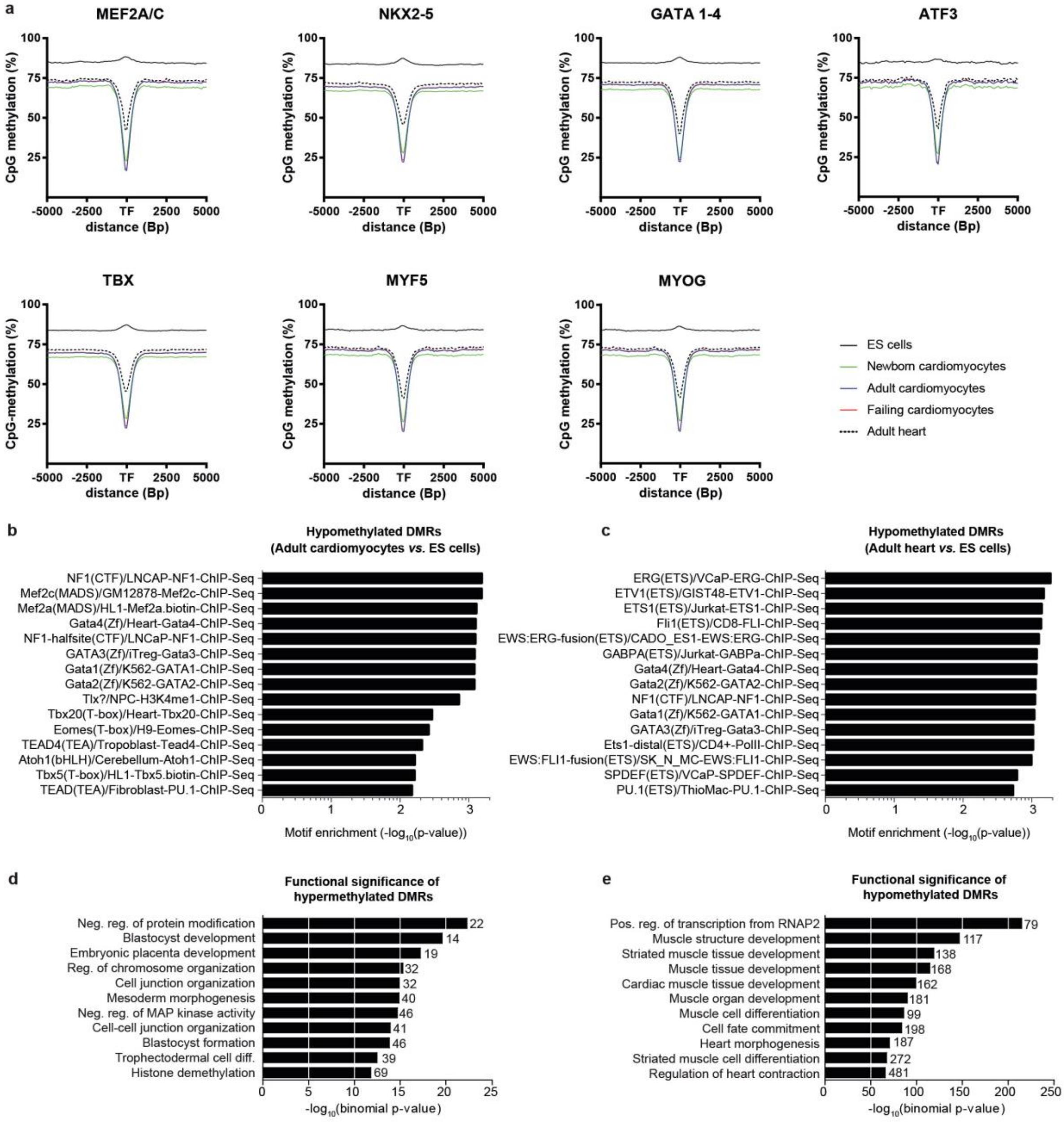

Suppl. Fig. 7: a, CpG methylation of 5 kbp flanking regions of cardiac transcription factor (TF) binding motifs identified in DMRs which were hypomethylated in adult cardiomyocytes *vs.* ES cells (TF factor motifs highlighted in bold in Fig. 1f). **b-c,** Transcription factor motif enrichment in hypomethylated DMRs of cardiomyocytes (b) and hearts (c) as compared with ES cells. Graphs display the 15 most significantly enriched motifs identified by HOMER (hypergeometric test). **d-e,** GO biological analysis of genes with proximal (-5 - +1 kbp) or distal (up to 50 kbp) DMRs by GREAT. Functional significance of hyper- (d) or hypomethylated (e) DMRs in adult cardiomyocytes as compared with ES cells. Numbers of genes per group are indicated next to the columns (binomial test).

Supplementary Figure 8: Gene ontology analysis of hypermethylated gene bodies in adult cardiomyocytes *vs.* ES cells

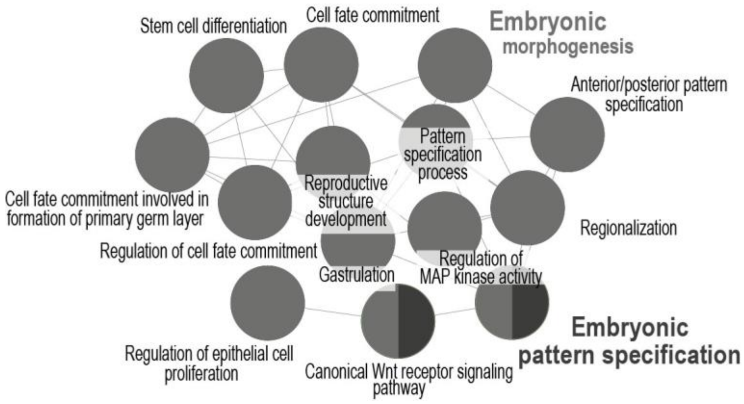

Suppl. Fig. 8: Gene ontology analysis of genes which were hypermethylated (from Fig. 2, group I) in adult cardiomyocytes *vs.* ES cells. *P* value per GO term $< 10^{-3}$ for hypermethylated genes, hypergeometric test, Bonferroni step down correction.

Supplementary Figure 9: Gene ontology analysis of hypomethylated gene bodies in adult cardiomyocytes *vs.* ES cells

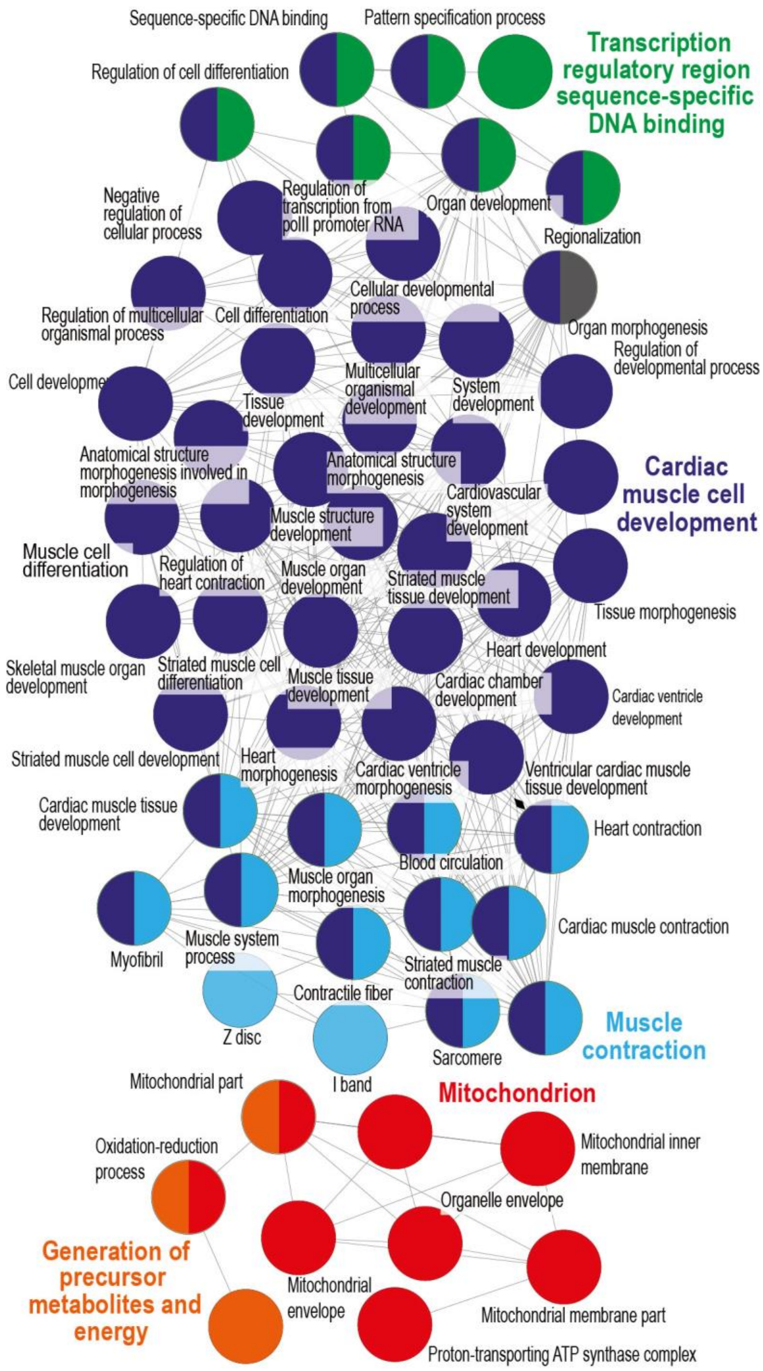

Suppl. Fig. 9: Gene ontology analysis of genes which were hypomethylated (from Fig. 2, group II) in adult cardiomyocytes *vs.* ES cells. *P* value per GO term $< 10^{-13}$ for hypomethylated genes, hypergeometric test, Bonferroni step down correction.

Supplementary Figure 10: Gene ontology terms of genes with hypomethylated gene bodies in adult *vs.* newborn cardiomyocytes

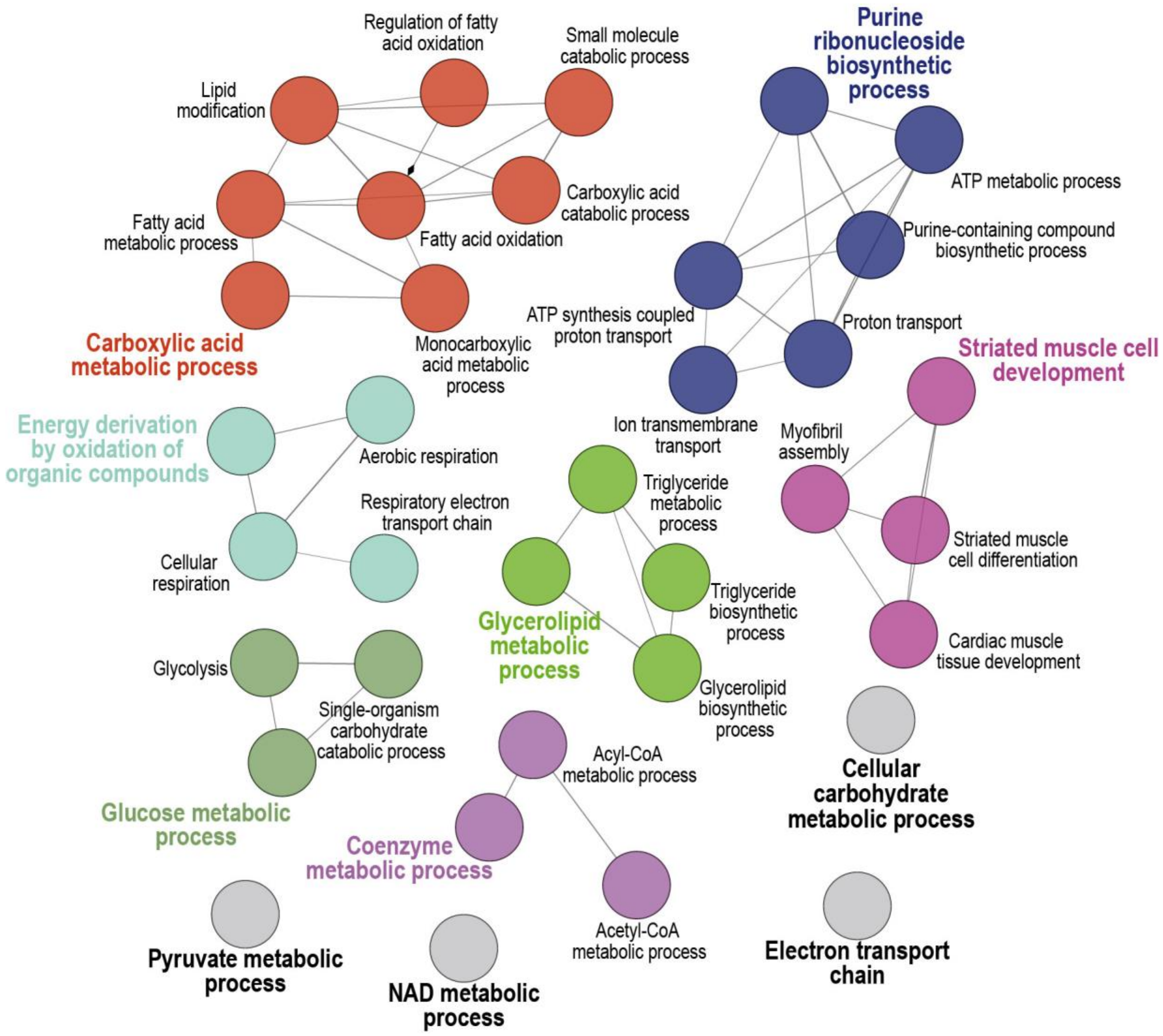

Suppl. Fig. 10: Gene ontology analysis of hypomethylated genes in adult *vs.* neonatal cardiomyocytes (P value per GO term < 0.001, hypergeometric test, Bonferroni step down correction).

Supplementary Figure 11: Gene ontology terms of genes with hypermethylated gene bodies in adult *vs.* newborn cardiomyocytes

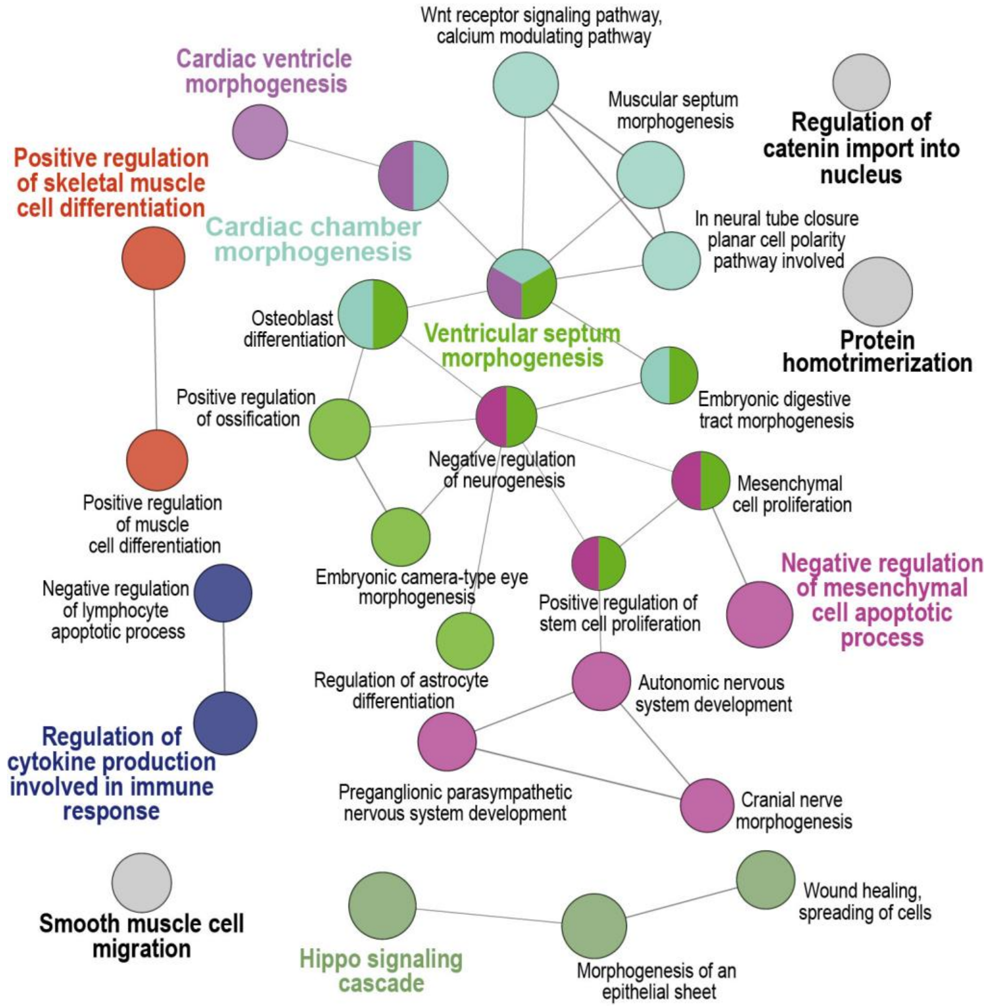

Suppl. Fig. 11: Gene ontology analysis of hypermethylated genes in adult *vs.* neonatal cardiomyocytes (*P* value per GO term < 0.05, hypergeometric test, Bonferroni step down correction).

Supplementary Figure 12: EZH2 binding and H3K27me3 of genes with low genic CpG methylation in cardiomyocytes

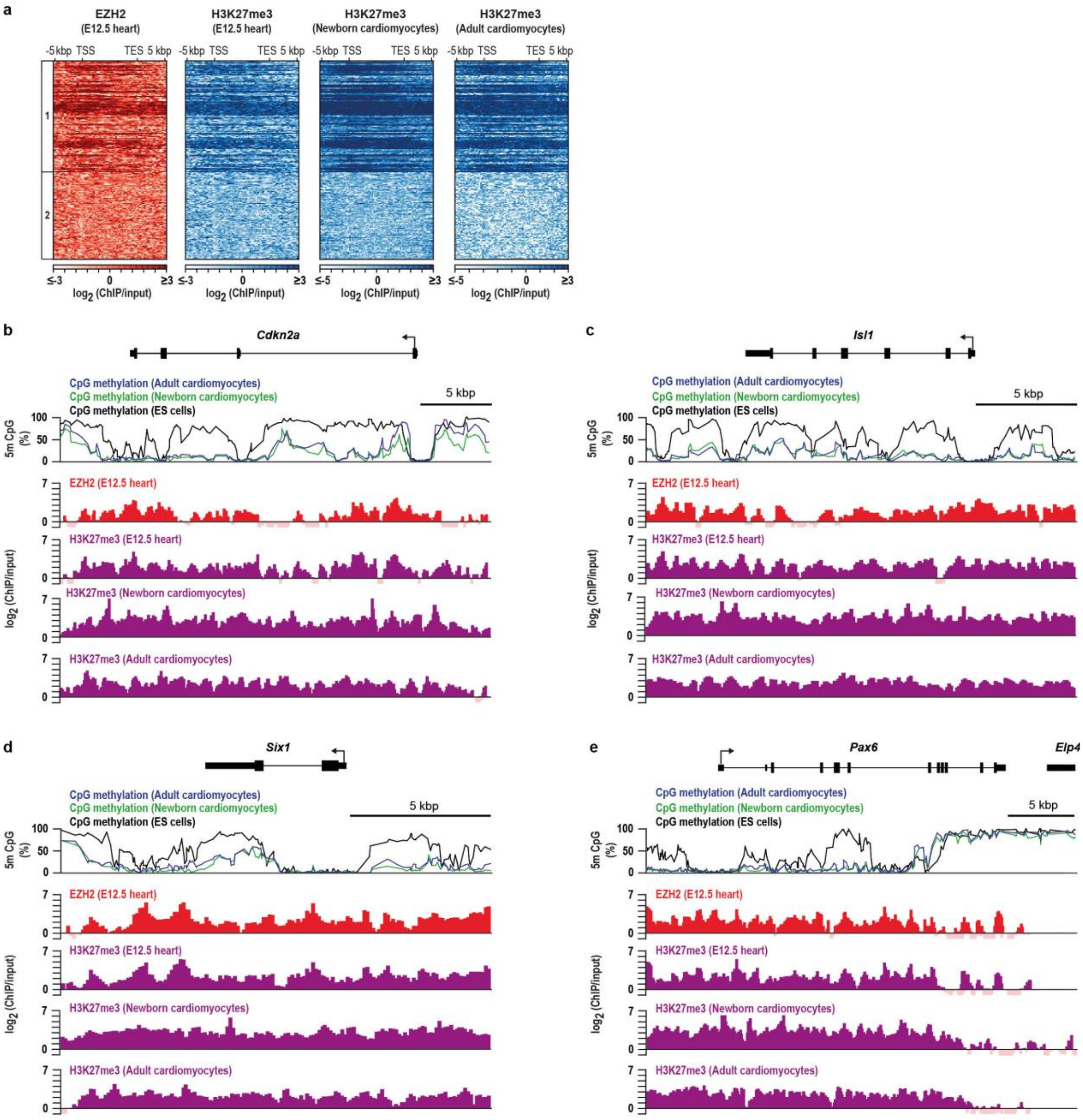

Suppl. Fig. 12: a, Heat maps of EZH2 binding and H3K27me3 marks for different developmental stages of genes which are demethylated in cardiomyocytes as compared with ES cells. Shown are results of E12.5 hearts[1], newborn and adult cardiomyocytes. Displayed genes have either very low (< 1 FPKM, from Fig. 5, group 1) or high expression (> 250 FPKM, from Fig. 5, group 2) in adult cardiomyocytes. For comparison, adult cardiomyocyte H3K27me3 data are reproduced from Fig. 5c. **b-e,** IGV traces of four representative loci. Displayed are CpG methylation as well as H3K27me3 and EZH2 enrichments. E12.5 data are reanalyzed from He et al.[1]. Abbreviations: TES, transcription end site; TSS, transcription start site; 5m CpG, CpG methylation.

Supplementary Figure 13: Time course of DNA methylation of two genes which are hypermethylated in adult *vs.* newborn cardiomyocytes

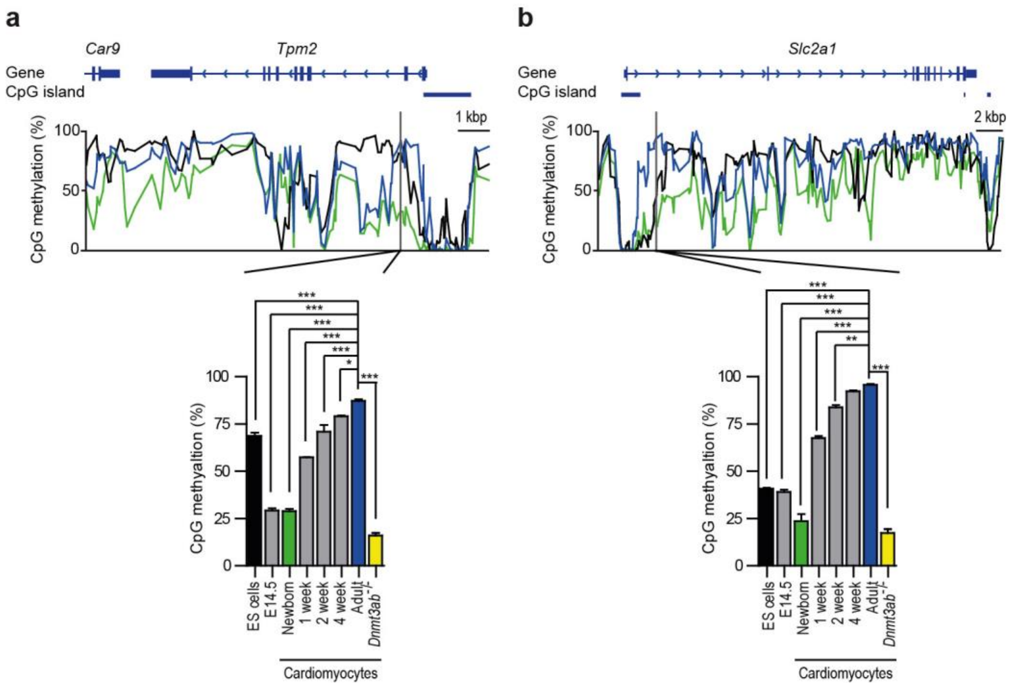

Suppl. Fig. 13: a, b, Upper traces, IGV (Integrative Genomics Viewer) traces of CpG methylation of tropomyosin 2 (*Tpm2,* a) and glucose transporter GLUT1 (*Slc2a1*, b) in ES cells, newborn and adult cardiomyocytes. Lower graphs, the time course of DNA methylation was analyzed by pyrosequencing during foetal and postnatal development and after deletion of *Dnmt3a/b*. Data are mean values ± s.e.m., *n* = 3-4 hearts per group, * *P* < 0.05, ** *P* <0.01, ****P* < 0.001 *vs.* adult, ANOVA, Bonferroni post hoc test). E, embryonic day.

Supplementary Figure 14: Analysis of DNA methylation of cell cycle-associated genes during postnatal development of cardiomyocytes

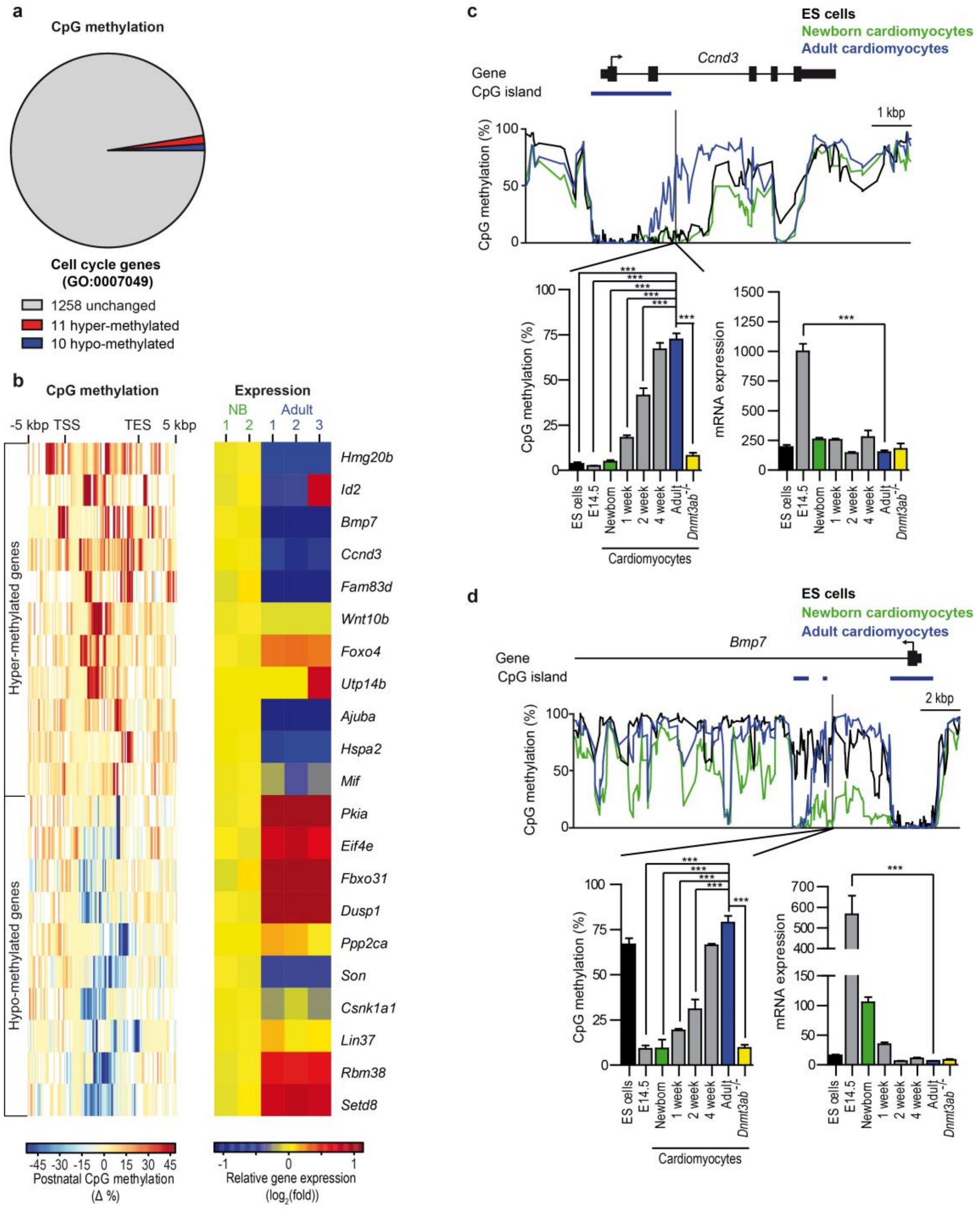

Suppl. Fig. 14: a, Pie chart of postnatal differentially methylated cell cycle genes (GO:0007049). Genes harboring DMRs covering > 5% of the entire gene body were regarded as differentially methylated. **b,** Heatmaps of CpG methylation and gene expression changes of postnatally differentially methylated cell cycle genes (*n* = 2 newborn, 3 adult samples). **c, d,** Upper panels, representative IGV traces of DNA methylation in newborn and adult cardiomyocytes and ES cells. Lower panels, the time course of CpG methylation and gene expression was analyzed by pyrosequencing during foetal and postnatal development and after deletion of *Dnmt3a/b* (data are mean values ± s.e.m., *n* = 3-4, ***$P < 0.001$ *vs.*

adult, ANOVA, Bonferroni post hoc test). Abbreviations: E, embryonic day; TSS, transcription start site; TES, transcription end site.

Supplementary Figure 15: Analysis of expression, H3K27me3 marks and DNA methylation and of cell cycle-associated genes during postnatal development of cardiomyocytes

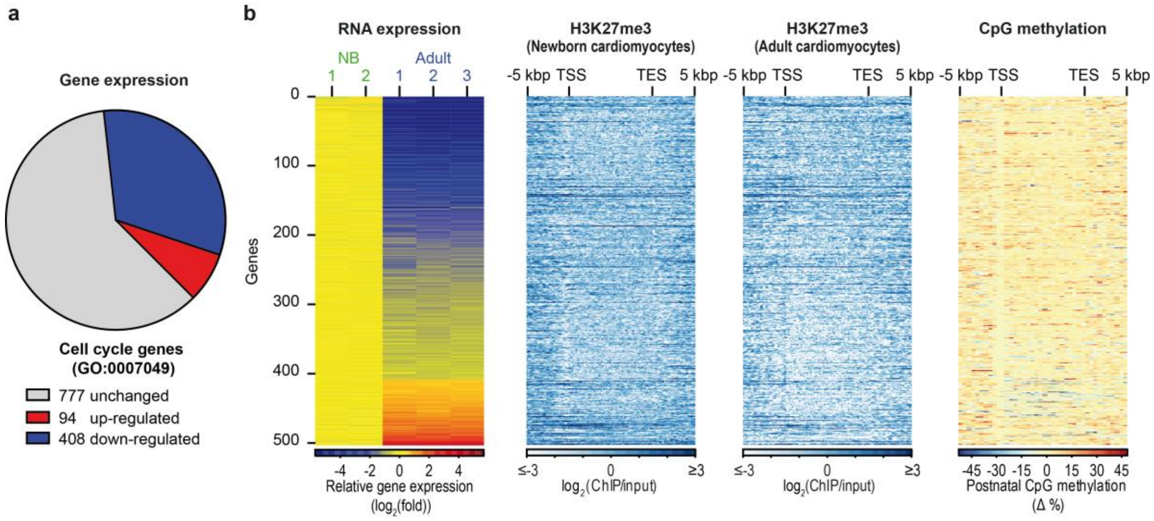

Suppl. Fig. 15: a, Pie chart of postnatal differentially regulated cell cycle genes ($P < 0.05$, > 1.5 fold, Student's t-test). **b,** Heat maps of postnatal gene expression and CpG methylation changes as well as levels of H3K27me3 binding in newborn and adult cardiomyocyte genes ($n = 2$ newborn, 3 adult samples). Abbreviations: TSS, transcription start site; TES, transcription end site

Supplementary Figure 16: Experimental murine heart failure model

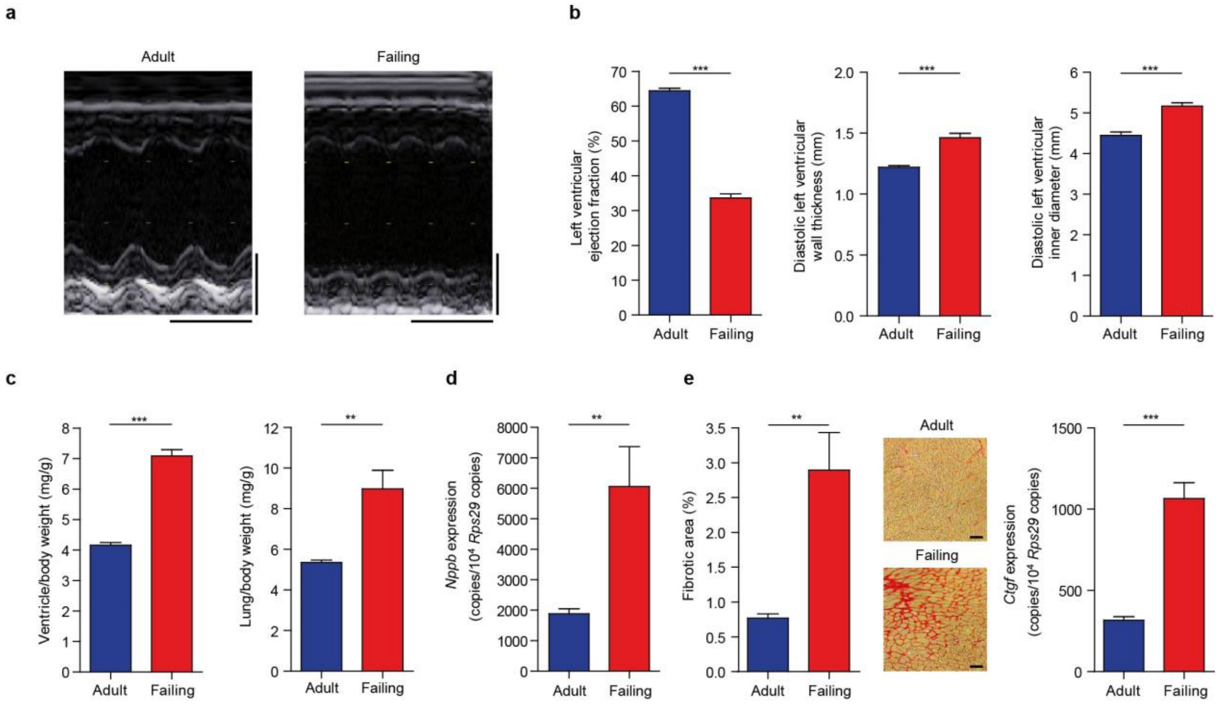

Suppl. Fig. 16: Chronic cardiac pressure overload in mice after 3 weeks of transverse aortic constriction was assessed by echocardiography (a, representative M-mode tracings, vertical bar 2 mm, horizontal bar 200 msec) and led to reduced left ventricular ejection fraction, increased diastolic wall dimensions and chamber dilatation (b, *n* = 10-11), cardiac hypertrophy and pulmonary edema (c, *n* = 10-11), increased expression of brain natriuretic peptide (d, *Nppb*, *n* = 4-5) and increased cardiac fibrosis (Sirius red staining, bar 50 µm) and connective tissue growth factor (*Ctgf*) mRNA expression (e, *n* = 4-5). Data are shown as mean values ± s.e.m.; ****P* < 0.001, ***P* < 0.01, Student's t-test.

Supplementary Tables

Supplementary Table 1: MethylC-seq sequencing statistics.

Group	Libraries	Lanes	Biological replicates	Number of assessed CpGs	Mean coverage per CpG
Newborn cardiomyocytes	3	3 (100 PE)	10	21440383	29
Adult cardiomyocytes	3	5 (100 PE)	16	21489071	38
Failing cardiomyocytes	3	5 (100 PE)	18	21494224	38

Experimental details of methylC-seq experiments. PE, paired-end reads.

Supplementary Table 2: ChIP-seq sequencing statistics.

Histone modification	Replicate 1 (No. of reads)	Replicate 2 (No. of reads)	Peaks (No.)
H3K4me1 (Adult)	34136245	27433336	197175
H3K4me3 (Adult)	7112750	888472	29745
H3K27me3 (Adult)	17186311	8801007	14539
H3K27ac (Adult)	37254499	10367429	100133
Input (Adult)	76866427	66920579	
H3K27me3 (Newborn)	13492727	17480255	22352
Input (Newborn)	62552105		

Number of uniquely mapped reads for ChIP-seq experiments and MACS2 peaks. Pearson correlation coefficient between replicates was > 0.9 for all assessed modifications and input.

Supplementary Table 3: Expression of genes with low genic CpG methylation after cardiac ablation of EZH2.

	**E12.5 heart**[1]	**Adult right ventricle**[2]
**Gene**	RNA expression (*Ezh2* mutant/control)	RNA expression (*Ezh2* mutant/control)
Cdkn2a	108.6	27.6
Six1	33.6	17.1
Isl1	12.3	n.d.
Pax6	11.5	n.d.
Clec4n	n.d.	3.2
Shox2	3.0	n.d.
Hoxb3	2.9	n.d.
Pitx2	2.2	n.d.
Ezh2 mutant	$Ezh2^{fl/fl}$ x $Nkx2\text{-}5^{Cre/+}$	$Ezh2^{fl/fl}$ x $Mef2cAHF^{Cre}$
Control	$Ezh2^{fl}$ x $Nkx2\text{-}5^{Cre/+}$	$Ezh2^{fl/fl}$

Cardiomyocyte-specific deletion of the *Ezh2* gene led to increased gene expression in E12.5 heart or adult right heart (regulated genes FPKM > 1; > 1.5 fold; $P < 0.05$; n.d. not detectable, i.e. FPKM < 1 in all samples). Data are derived from published RNA-seq data sets[1,2].

Supplementary References

1 He, A. *et al.* Polycomb repressive complex 2 regulates normal development of the mouse heart. *Circ Res* **110**, 406-415 (2012).

2 Delgado-Olguin, P. *et al.* Epigenetic repression of cardiac progenitor gene expression by Ezh2 is required for postnatal cardiac homeostasis. *Nat Genet* **44**, 343-347 (2012).
